# EDF1 accelerates ganglioside GD3 accumulation to boost CD52-mediated CD8^+^ T cell dysfunction in neuroblastoma

**DOI:** 10.1186/s13046-025-03307-9

**Published:** 2025-02-04

**Authors:** Di Li, Meng Li, Zhenjian Zhuo, Huiqin Guo, Weixin Zhang, Yile Xu, Hai-Yun Wang, Jiabin Liu, Huimin Xia, Huiran Lin, Jue Tang, Jing He, Lei Miao

**Affiliations:** 1 Department of Pediatric Surgery, Guangdong Provincial Key Laboratory of Research in Structural Birth Defect Disease, Guangzhou Women and Children’s Medical Center, Guangzhou Institute of Pediatrics, Guangzhou Medical University, Guangzhou, 510623 Guangdong China; 2 Laboratory Animal Center, School of Chemical Biology and Biotechnology, Peking University Shenzhen Graduate School, Shenzhen, 518055 Guangdong China; 3 Department of Pathology, Guangzhou Institute of Pediatrics, Guangzhou Women and Children’s Medical Center, Guangzhou Medical University, Guangdong Provincial Clinical Research Center for Child Health, National Children’s Medical Center for South Central Region, No. 9 Jinsui Road, Guangzhou, 510623 Guangdong China; 4 Laboratory Animal Management Office, Shenzhen Institutes of Advanced Technology, Chinese Academy of Sciences, Shenzhen, 518055 Guangdong China

**Keywords:** EDF1, Neuroblastoma, CD8^+^ T cell dysfunction, LacCer metabolism, GD3

## Abstract

**Background:**

Heterogeneous clinical features and prognosis in neuroblastoma (NB) children are frequently dominated by immune elements. Dysfunction and apoptosis in immune cells result from the exposure to continuous tumor-related antigen stimulation and coinhibitory signals. To date, key factors pointing to the restriction of NB-specific CD8^+^ T cells remain elusive.

**Methods:**

We performed bulk-RNA sequencing and lipidomic analyses of children with mediastinal NB. Bioinformatics analysis and biological validation were applied to uncover the underlying mechanism.

**Results:**

Three subtypes were identified using nonnegative matrix factorization (NMF), among which we highlighted an apoptotic status of infiltrated CD8^+^ T cells, along with the highest CD52 and EDF1 expression in Cluster3 (C3) subtypes. It was verified that high EDF1 expression in NB cells led to Lactosylceramide (LacCer) accumulation, as well as downstream ganglioside-GD3, which subsequently increased the expression of CD52 and immune checkpoint genes, chemotaxis, and apoptosis-related events in activated CD8^+^T cells. Mechanistically, EDF1 was recruited as a coactivator to form the NF-κB/RelA/EDF1 complex, which further prevented the promoter region methylation of ST8SIA1, to elevate its transcription.

**Conclusion:**

These findings characterize abundant GD3 in NB cells, which regulated by the EDF1/RelA/ST8SIA1 axis, is responsible for CD8^+^ T cell dysfunction. Inhibition of EDF1 may reduce suppressive factors and prevent immune escape of NB cells. Modulating NB-associated GD3 levels through metabolic intervention is beneficial for tuning the depth and duration of responses to current NB therapies. The integration of transcriptomic and lipidomic data offers a more comprehensive understanding of the interaction between LacCer metabolites and the immune status in NB.

**Graphical Abstract:**

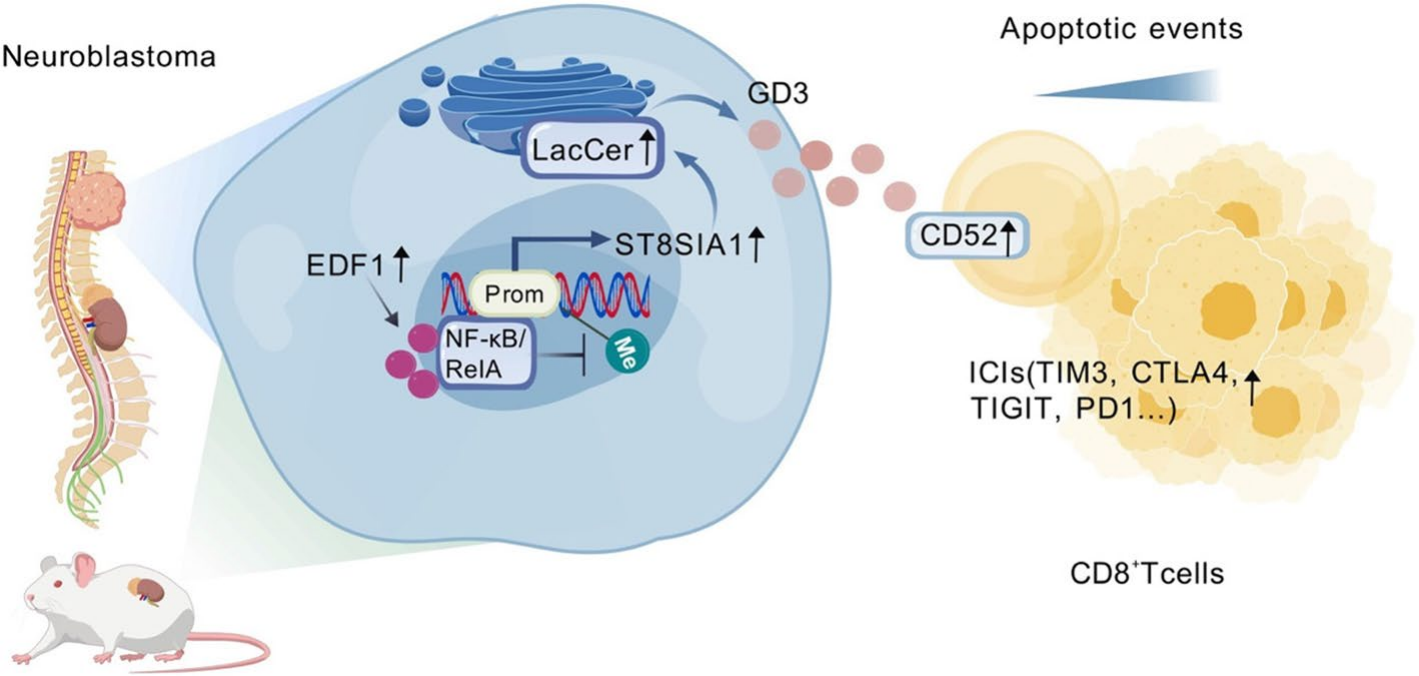

**Supplementary Information:**

The online version contains supplementary material available at 10.1186/s13046-025-03307-9.

## Importance of the study

We highlight EDF1 in NB facilitates LacCer/GD3 metabolism and leads to CD8^+^ T cell dysfunction. GD3 is a highly potential marker for monitoring the efficiency of NB therapies.

## Introduction

Neuroblastoma (NB) is characterized as an high heterogeneous extracranial solid tumor with low mutational burden [1], it maintains immunosuppressive microenvironment by downregulating MHC class-I and secreting inhibitory factors, which subsequently hampers the activation of effector cells and the infiltration of immune cells [2–5].

Persistent stimulation by tumor-related antigens and coinhibitory signals contributes to immune cell dysfunction, as evidenced by reduced tumoricidal cytokines, inhibited immune cell proliferation, and elevated expression of inhibitory receptors [6]. Although studies have reported a beneficial prognostic role of cytotoxic T-cell infiltration in high-risk NB [7], the reactivity of tumor infiltrating lymphocytes (TILs) towards NB is always hindered by immune checkpoints [8–10]. Up-regulated levels of immune checkpoint genes (*TIM3*, *CTLA4*, *TIGIT*, *PD1*) were also found in NB with high cytotoxic signatures, indicating chronic stimulation of T cells with their cognate antigens may lead to an exhausted state [5].

Lactosylceramide (LacCer) is a member of glycosphingolipids (GSLs), which are involved in cell membrane integrity and several cellular processes, such as apoptotic and inflammatory programs [11–13]. LacCer primarily serves as a backbone structure to gangliosides and other complex glycosphingolipids [14]. Gangliosides is anchored on the outer surface of the plasma membrane, they serve as bioactive lipid mediators and play a crucial role in a wide range of cellular functions [15–17]. aberrant accumulation of gangliosides-GD3 and -GD2 are associated with poor prognoses in melanoma and NB [18–20], partly occurs by their suppressive effects on lymphocytes [21]. Although immunotherapy targeting GD2, has significantly improved outcomes for NB patients [22, 23], the exhaustion of effector T cells hindered their persistence and cytotoxic activity. To date, there is a lack of recognition between LacCer-derived gangliosides and their immunological roles during NB progression. Herein, we present a translational investigation into GD3-associated NB progression and its impacts on CD8^+^ T cell dysfunction, with the aim of sustaining and enhancing tumor-killing activities in NB patients.

## Materials and methods

### Mediastinal neuroblastoma (MNB) sample collection and data processing

A total of 89 surgically resected MNB tumors with clinical and demographic characteristics (Table S1) were collected for transcriptome sequencing. Ethical approval was obtained from the institutional ethics committee of Guangzhou Women and Children’s Medical Center (2023-120A01). The raw sequence RNAseq data was deposited in NCBI’s Gene Expression Omnibus (GSE285546). Our study was conducted strictly to meet the ethical standard of the World Medical Association Declaration of Helsinki. No interventional experiments were performed throughout the whole study.

Total RNA from MNB samples, as well as EDF1-altered NB cells and cocultured PBMCs were extracted [24] for cDNA library construction and sequencing using an Illumina NovaSeq 6000 platform. The count data was generated and transformed for subsequent analysis.

### Nonnegative matrix factorization (NMF) classification of MNB tumors

We used the “NMF” (v0.23.0) [25] R package to depict co-expression gene characteristic within the transcriptomic matrix. Normalized data was subjected to unbiased classification with decomposit NMF algorithm, and typed with a rank set range from 2 to 10. The “brunet” method was selected, and nrun was set to 100. The optimal number of clusters was determined by dispersion, cophenetic and RSS coefficients. The “Rtsne” package (v0.16) was applied to verify the feasibility of the NMF typing results [26]. Principal component analysis (PCA) of standardized data among the clusters was performed by the “prcomp” function of “stats” (v4.2.1) [27] (using variance Stabilizing Transformation method), and visualized by the “pheatmap” (v1.0.12) [28]. The “ggstatsplot” (v0.11.0) [29] and “corrgram” (v1.14) [30] R packages were used to perform correlation analysis in each MNB sample.

### Differentially expressed genes (DEGs) and functional enrichment analysis among clusters

We implemented the “limma” R package (v3.52.4) [31] and “DESeq2” R(1.38.3) [32] package to analyze the DEGs between identified subtypes and groups according to the median of indicated gene expression, and visualized by “Enhanced Volcano” (v3.18) [33] R packages. The “VennDiagram” (v1.7.3) [34] R package was used for screening DEGs in both C3 *v.s.* C2 and C3 *v.s.* C1.

To obtain biological pathways of gene set enrichment, the “clusterProfiler” R package (v4.4.4) [35] was applied for gene ontology (GO) and Kyoto Encyclopedia of Genes and Genomes (KEGG), as well as gene set enrichment analysis (GSEA) based on the total gene expression profile. The results were ranked and plotted by P value or the functional enrichment score and visualized by the “ggplot2” (v3.4.2) [36] package. To elucidate variations in single gene expression across various clinical settings, boxplots were generated using the “ggpubr” (v0.6.0) [37] package in R, displaying log2-transformed expression levels. A normalized *P* value < 0.05 was considered statistically significant.

### Difference enrichment scores of major cell components in MNB tumors

According to the gene signatures of 22 kinds of immune cells, the CIBERSORT deconvolution algorithm was used to assess the degree of immune cell infiltration in the three clusters by the Wilcoxon test [38], which was run in absolute mode using the LM22 signature over 1000 permutations. Additionally, we manually generated a list of refined 32-component signatures in the tumor microenvironment (TME) (Table S2), which were integrated by an “immunophenoscore” model of 28 major immune cells from Dong *et al.* [39], as well as cellular characteristics from Bagaev *et al.* [40]. These components covered the major immune celltype, immune-related properties (checkpoint molecules, coactivation molecules), stromal compartments (cancer-associated fibroblasts, endothelium, angiogenesis) and tumorigenic characteristics (EMT, matrix remodeling). Based on transcriptomic analyses in MNB, the “GSVA” package (v1.46.0) [41] and random walk algorithm of single sample gene set enrichment analysis (ssGSEA) were applied to evaluate enrichment scores of major components in each cluster of MNB.

### Survival analysis for screening target genes

Based on the median gene expression counts downloaded from GSE49710, the prognostic value of a single gene was evaluated by OS and PFS. We used the “survival” R package (v3.5–5) [42] with the optimal cutoff value calculated using the “surv_cutpoint” function, followed by the “survminer” R package (v0.4.9) [43] to plot Kaplan‒Meier survival curves.

### Lipidomic analysis

Lipids in 42 MNB samples were extracted from chloroform methanol mixed solution (2:1) as soon as grinding. After concentration in vacuum, samples were dissolved in isopropanol and filtered through a 0.22 μm membrane to obtain the prepared samples for quality control (QC) and LC–MS detection. The chromatographic and mass spectrometry parameters were preset by PANOMIX.Inc. Differentially abundant metabolites were obtained based on bioformatic analysis flow for lipidomic raw data.

### Cell culture and isolation and reagents

The human NB cell lines SK-N-BE, SK-N-AS, IMR32, SH-SY5Y and SK-N-SH were obtained from the American Type Culture Collection (ATCC, Manassas, VA, USA). SK-N-BE cells were maintained in a mixture of EMEM and F12 (Gibco, Thermo Fisher Scientific, Dublin, Ireland) (1:1). SK-N-SH cells were maintained in EMEM, and SK-N-AS, SH-SY5Y and IMR32 cells were maintained in RPMI-1640. All types of media consisted of 10% fetal bovine serum (FBS) and 1% penicillin/streptomycin. Cells were all incubated at 37 °C with 5% CO_2_ and tested without mycoplasma contamination.

Human purified CD8^+^ T cells were isolated from peripheral blood mononuclear cells (PBMCs) by anti-CD8-positive selection kit and cultured in T-cell expansion medium (ImmunoCult-XF supplemented with CD3/CD28/CD2 T cell activator and human IL-2) for stimulation and expansion. Reagent details are shown in Table S3.

### Cell transfection

Human cDNA of human full-length EDF1 was cloned and ligated into a pcDNA3.1 vector. The shRNA sequences targeting human EDF1 were inserted into the vector pDKD-CMV-Puro-U6-shRNA. Cells were transfected by using Lipofectamine™ 3000. The scrambled shRNA sequence was used as a control for EDF1 knockdown, while the empty vector (EV) was used as a control for EDF1 overexpression. All plasmids were provided by OBiO Technology Inc. (Shanghai, China).

Small interfering RNAs (siRNA) targeting EDF1 and ST8SIA1 were separately used to transiently knockdown the indicated genes (RiboBio Co., Ltd, Guangzhou, China), and a control transfected with scrambled sequence was used in the study. The sequences of all the siRNA and shRNA are shown in Table S4.

Human full-length CD52 (AdCD52) cDNA was packaged into adenoviruses (pADV-U6-shRNA-CMV-MCS) by OBiO Technology Inc. for CD8^+^ T transfection. The knockdown or overexpression efficiency was validated by analyzing the mRNA and protein levels of targeted genes.

### Chromatin immunoprecipitation (ChIP) and ChIP-PCR

DNA fragments in NB cells were immunoprecipitated with NF-κB p65/RelA according to the protocol of a Simple ChIP kit. The protein‒DNA complexes were further reacted with Simple CHIP qPCR Master Mix for quantitative real-time PCR assays. Primers targeting DNA-binding sites are shown in Table S5.

### Co-IP assay

EDF1-binding proteins were immunoprecipitated by anti-EDF1 antibody, followed by mass spectrometry. The identified peptides are shown in Table S6. Lysates immunoprecipitated by anti-EDF1 and anti-NF-κB p65/RelA antibodies were subjected to Co-IP detection as described previously [44] to verify the combination with each other. Anti-rabbit and mouse IgG antibodies were used as negative controls.

### Immunofluorescence (IF)

Briefly, MNB tumor slices, as well as NB cells, were fixed and permeabilized, followed by staining simultaneously with anti-human CD52 and CD8 antibodies for tumor slices, as well as anti-ST8SIA1 and anti-GD3 antibodies for NB cells. They were further incubated with anti-mouse or rabbit Alexa Fluor secondary antibodies. The nuclei were subsequently visualized with 4',6-diamidino-2-phenylindole (DAPI) staining. For CD8^+^ T cells, after adhering to Matrigel-coated confocal chambers and subjected to GD3 (0, 50, 100 µg/ml) for 1 h at 4 °C, CD8^+^ T cells were fixed, permeabilized and immunostained with anti-GD3 and Anti-COX2 followed by accordant secondary antibodies. Images of the cells were captured by a confocal microscope (Leica SP8, Germany).

### Immunohistochemistry (IHC)

NB tissues and mouse-derived tumor samples were fixed and embedded for IHC staining as previously described [44]. After blockade, the sections were separately incubated with rabbit anti-EDF1 antibody and rabbit anti-Ki67 antibody at 4 °C overnight, hybridized with biotinylated anti-rabbit/mouse immunoglobulin at room temperature and visualized by diaminobenzidine (DAB). Three to five representative fields of each section were captured and analyzed. The average positive ratio was defined by the symbol and color, and was calculated.

### Cellular proliferation assays

The viability of NB cells was determined by CCK-8 assay. Briefly, NB cells were plated in 96-well plates overnight and treated as indicated. The cell viability index was obtained by calculating the absorbance of each well at 450 nm after incubation with CCK-8 for 2 h.

For the 5,6-carboxyfluorescein diacetate, succinimidyl ester (CFSE) staining, cells were stained with 0.5 µM CFSE for 20 min at 37 °C, washed with PBS for 3 times and treated as indicated, followed by flow cytometric detection.

### Cell migration and chemotaxis

For the migration assay, NB cells (5 × 10^4^ per well) with 1% FBS were seeded in the upper chambers of a 24-well Transwell inserts (Corning, NY, USA) and then exposed to medium consisting of 10% FBS in the lower chambers (inserts with 8 μm pore size). Cells migrated to the lower surface of the inserts were counted after 12 h and analyzed by ImageJ software.

For the CD8^+^ T cell chemotaxis, purified CD8^+^ T cells (1 × 10^6^/well) were seeded in the upper chambers of a 24-well Transwell inserts (Corning, NY, USA) and exposed to medium containing the indicated NB cell-based conditioned medium (CM) in the lower chambers (inserts with 0.4 μm pore size) for 12 h. The chemotaxis activity was analyzed by counting the number of migrated cells.

### Cell apoptosis and ROS detection

CD8^+^ T cells and NB cells were harvested and washed with PBS and then resuspended in staining buffer containing propidium iodide (PI) and Annexin-V-AF647 (ES Science, Shanghai, China). The percentages of cells undergoing apoptosis were evaluated by flow cytometry.

The reactive oxygen species (ROS) staining was performed with a ROS Assay Kit. Cells were harvested and washed with PBS for 3 times, resuspended in PBS and incubated with DCFH-DA fluorescence probe for 1 h at 37 °C. After washing with PBS twice, the cells were analyzed by flow cytometry.

### Cellular nuclear and cytoplasmic extraction and immunoblotting

Total protein lysates were extracted from treated cells lysed with ice-cold radio immunoprecipitation assay (RIPA) buffer containing protease and phosphatase inhibitors. Proteins in the cellular cytoplasm and nucleus were extracted by the NE-PER Nuclear and Cytoplasmic Extraction Reagents Kit (Thermo Fisher Scientific, MA, USA). Cytoplasmic protein was extracted by Cytoplasmic Extraction Reagents I and II after vortexing and incubation, and nuclear protein was obtained with Nuclear Extraction Reagent using the same method. The amounts of proteins were quantified and incubated with primary antibodies, including anti-EDF1, anti-CD52, anti-NF-κB/RelA, anti-β-actin and anti-histone H3 antibodies. All bands were visualized by enhanced chemiluminescence.

### Quantitative real-time PCR (qRT‒PCR)

Using an RNA extraction kit, total RNA was obtained, reversely transcribed to cDNA and quantified by qRT-PCR (ABI Q6 System, Applied Biosystems). The mRNA level was reflected as the ΔCt value. After normalization, the fold changes were calculated via the 2^−ΔΔCt^ comparative method. The sequences of the specific primers are shown in Table S5.

### Flow cytometry analyses for CD52 and GD3 staining

Purified CD8^+^ T cells were cocultured with the indicated NB cells for 48 h and then stained with APC-conjugated anti-human CD52 antibody for subsequent flow cytometry analysis. Alternatively, CD8^+^ T cells were exposed to GD3 and then fixed, permeabilized, and immunostained with anti-GD3 antibody followed by an Alexa 555-labeled secondary antibody. Data analysis was performed using FlowJo 10.4 Software (OR, USA).

### In vivo study

As previously described [44], orthotopic NB models were generated by injecting EDF1-altered SK-N-SH cells (5 × 10^5^ cells) expressing an inducible luciferase reporter into the left adrenal gland of immune-deficient B-NDG mice. All NB mice were randomly assigned into 3 groups (*n* = 10): NC + PBMC, ShEDF1 + PBMC and EDF1 + PBMC. After 2 weeks, equal size of tumors was screened and transplanted with 2 × 10^6^ human PBMCs once by tail vein injection.

For chemoresistance assay, equal size of tumors was randomly divided into 4 groups (*n* = 5 per group): NC + PBMC, ShEDF1 + PBMC, NC + PBMC + CDDP (2 mg/kg) and ShEDF1 + PBMC + CDDP (2 mg/kg). CDDP was administered twice per week followed by PBMC transplantation. Tumor size was monitored by luciferase imaging using In Vivo Bruker FX PRO. Luciferase intensity was captured and measured. The body weights were also tracked. All mice were euthanized when the luciferase intensity reached 10 [6]. Pieces of tumor tissues were fixed for histological and pathological analyses. All animal experiments met the standard and were approved by the laboratory animal center of Peking University Shenzhen Graduate School (ER-0042–011).

### Statistical analysis

All bioinformatics investigations were carried out using R software (version 4.3.1).

All western blots, immunofluorescence, flow cytometry data and IHC images are representative results of at least two independent biological replicates. Correlations between identified gene expression levels were determined with Pearson’s correlation analysis. All bar graphs show the means ± SDs. Statistical calculations were conducted from at least three independent experiments and analyzed by Student’s *t* test (unpaired, two-tailed) or one-way ANOVA using GraphPad Prism 9.4.1 software (GraphPad, La Jolla, CA, USA). A *P* value < 0.05 indicates statistical significance. In the figures, the following are used: *, *P* < 0.05; **, *P* < 0.01; and ***, *P* < 0.001.

## Results

### Defining immune cell infiltration subtypes of MNB by NMF typing

**Fig. 1:**
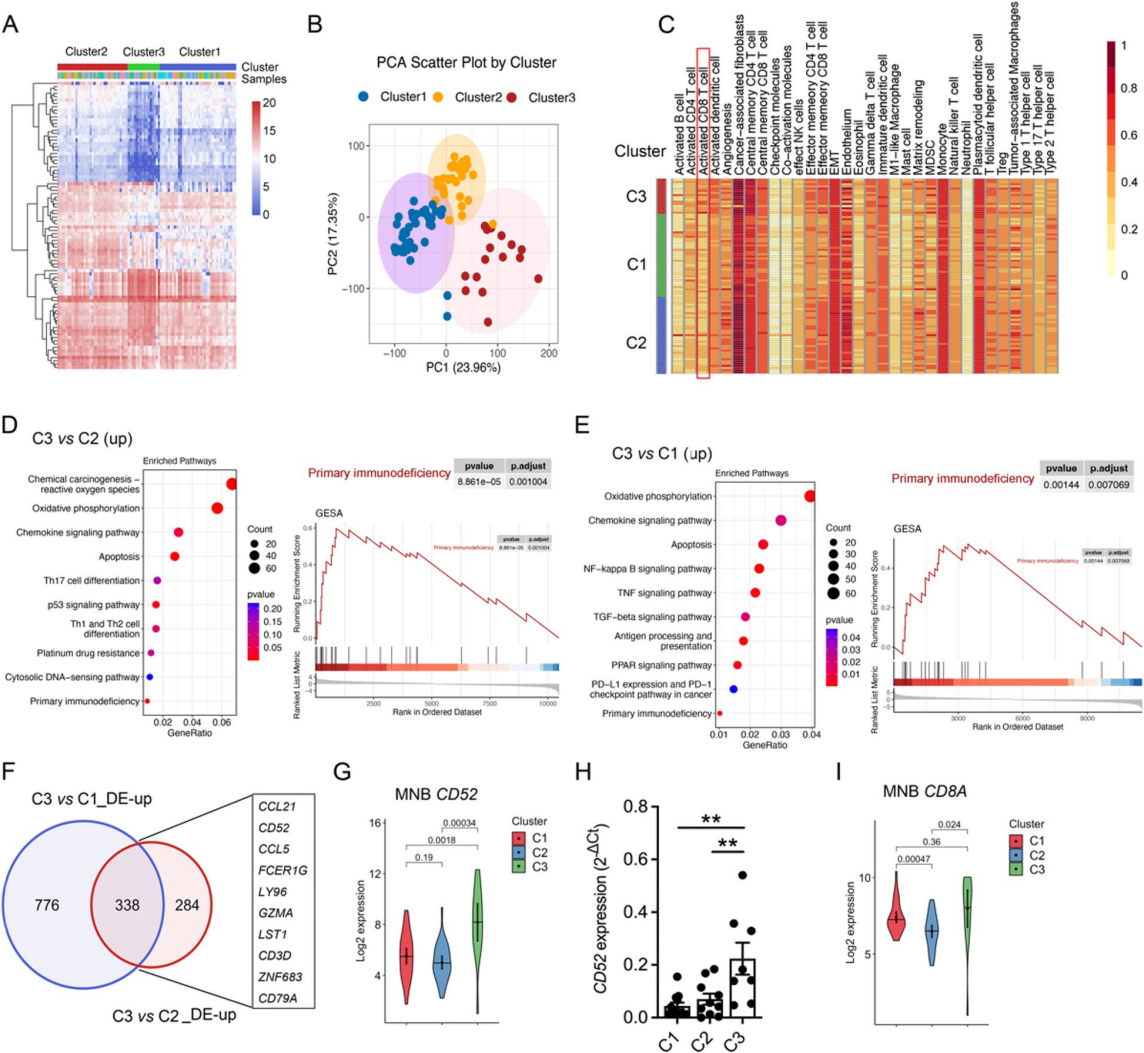
Identification of three immune-related clusters in the MNB transcriptional matrix. **A** Heatmap of transcriptomic data among the three clusters in the MNB samples for Rank = 3. **B** Principal component analysis (PCA) of standardize data among the clusters (using varianceStabilizingTransformation method). (C1: *n* = 38, C2: *n* = 35, C3: *n* = 16) (**C**) Evaluating the enrichment scores of major immune components in each MNB sample by ssGSEA. **D-E** KEGG and GSEA enrichment analyses of up-regulated DEGs in C3 *v.s.* C2 and C3 *v.s.* C1 (log2fold change > 2 and *P* value < 0.05), respectively. **F** Venn diagram of up-regulated DEGs in C3 *v.s.* C2 and C3 *v.s.* C1. **G, I** Log2 expression of *CD52* and *CD8A* in the transcriptional matrix across the three clusters. **H** mRNA expression of *CD52* in the three clusters. The data in G-I represent the mean ± SD. ^**^P < 0.01

Using unbiased non-negative matrix factorization (NMF) algorithm, maximization of cophenetic correlation values was used to provide weights to guide the optimal number for cluster reconstruction. We observed an inflection point at rank values of 3 to 4, along with a sharp decrease in both cophenetic and RSS coefficient curves (Fig. S1A, B). Therefore, MNB subtypes were decomposed into three non-negative matrices, and further validated through PCA dimensionality reduction analysis basing on the value of gene expression (Fig. 1A, B). Correlation analysis demonstrated a clear distinction among the three clusters: Cluster1(C1), Cluster2 (C2), and Cluster3 (C3) (Fig. S1C).

According to ssGSEA scores, Th2 cells were dominant part in C1. C2 was characterized by immunosuppressive cells (Treg, tumor-associated macrophages), along with stromal compartments, matrix modeling, EMT, and angiogenesis processes. Combing with Cibersort approaches, C3 was distinguished by significant immune cell infiltration, including activated CD4^+^ and CD8^+^ T cells, effector NK cells, and Th1 cells. Moreover, coactivation and checkpoint molecules were also highly expressed in C3 (Fig. 1C, Fig. S1D). Thus, we separated MNB into three types of microenvironments: (1) Infertile immune cell infiltration (C1); (2) Tumorigenic TME assembly by tumor-associated elements (C2); (3) Inflamed TME gathering by activated immune cells (C3). Notably, C3 had the fewest patients diagnosed at stage III/IV but exhibited the highest mortality rate. We hypothesized that the phenotypic characteristics of infiltrated immune cells in C3 mainly contributed to follow-up mortality.

### Functional enrichment among NMF types

After comparing the different gene expression profiles, we identified 1187 (C3 *v.s.* C2), 2430 (C3 *v.s.* C1), and 1574 (C2 *v.s.* C1) DEGs (|log2fold change|> 2 and *P* value < 0.05). Up-regulated DEGs in C2 *v.s.* C1 and C3 *v.s.* C1 shared common inflammatory-related pathways, while C3 *v.s.* C2 showed a predominant innate and adaptive immune pathways (Fig. 1D, E, Fig. S1E). The down-regulated DEGs were more concentrated on neuroactive ligand receptor-related processes (Fig. S 1F, G). Of note, C3 also significantly showed a positive association with primary immunodeficiency, PD-L1 expression and the PD-1 checkpoint pathway (Fig. 1D, Fig. S1G), raising the possibility that resident or chemotaxis CD8^+^ T cells in C3 may represent a dysfunctional continuum.

According to the comparation of C2 with C1, 338 up- and 365 down-regulated overlapping genes were identified (Fig. 1F, Fig. S1H, I). The top 10 candidates associated with chemotaxis and Pan-T cell stimulations and functions (*CCL21*, *CD52*, *CCL5*, *FCER1G*, *LY96*, *GZMA*, *LST1*, *CD3D*, *ZNF683* and *CD79A*) were screened (Fig. 1F). Of note, CD52, a GPI-anchored glycoprotein that rarely possesses an intracellular signaling domain, is mainly expressed on the cell surface of mature lymphoid cells and acts as a soluble form to suppress T-cell activation [45]. Regardless of stage classification (Fig. S1J), *CD52* exhibited the highest expression in C3 (Fig. 1G, H), so was *CD8A* (Fig. 1I). The data above enlightens us that the effect of CD52 on CD8^+^ T cells might be responsible for poor survival in C3.

### The prognostic feature of CD52 is predominantly associated with CD8^+^T cell infiltration

**Fig. 2:**
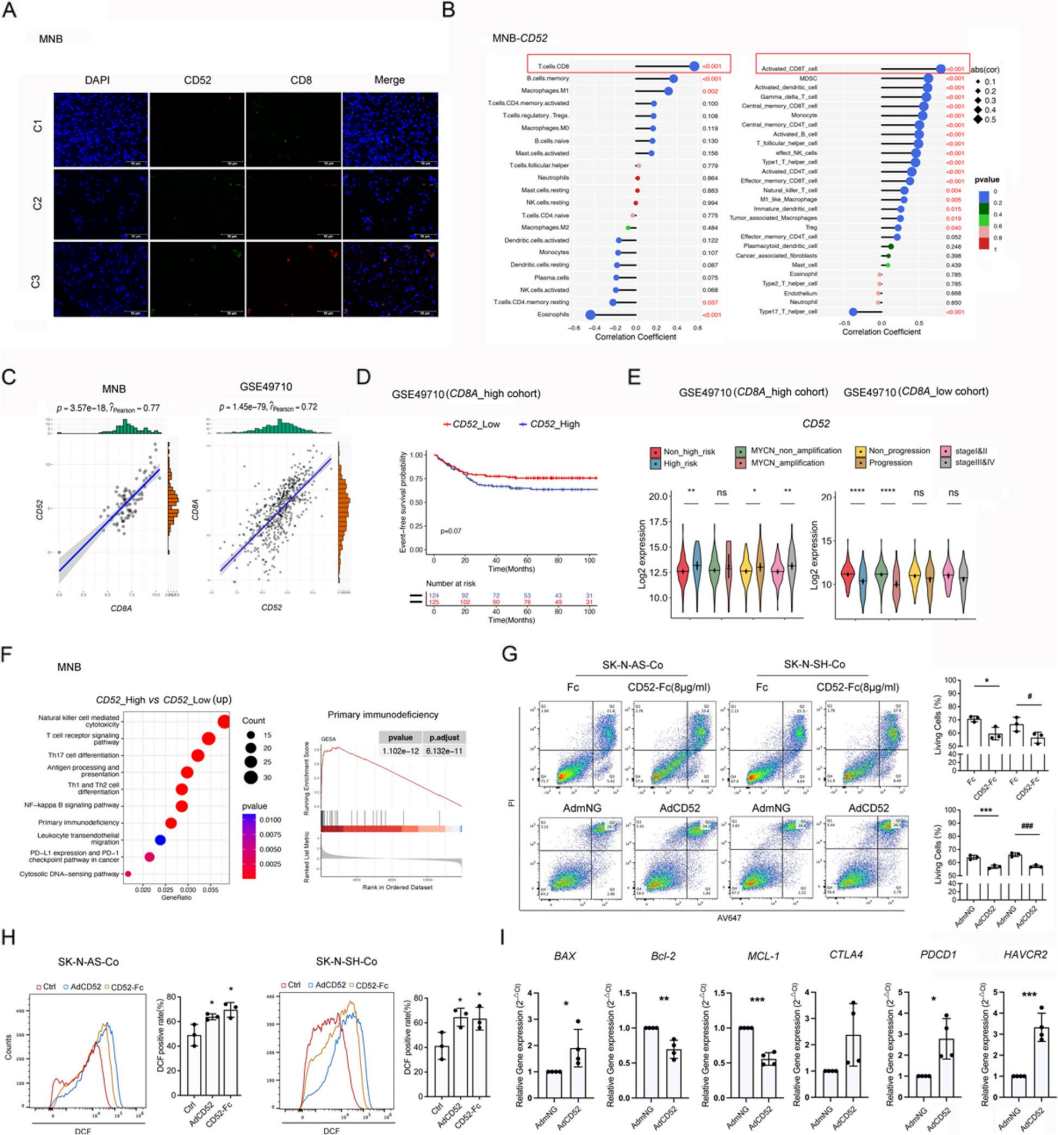
CD52 is positively associated with activated CD8^+^T cells in NB tumors and induces apoptotic events in the presence of NB cells.** A** Representative immunofluorescence staining images of CD8A (green) and CD52 (red) in each cluster of MNB samples (*n* = 4). DAPI (blue) was used to stain nuclei. The scale bar represents 50 µm. **B** Correlation analyses between *CD52* and immune components in MNB tumors. The size of the dots represents the correlated strength, and the color of the dots represents the accordant *P* value. **C** Correlation analysis showing the expression of *CD52* and *CD8A* in the MNB tumors and online dataset (GSE49710).** D** EFS of CD52 expression in the CD8A^high^ set from GSE49710. **E** Comparisons of CD52 Log2 expression across distinct clinicopathological characteristics of CD8A^high^ and CD8A^low^ NB tumors (GSE49710), independently. **F** KEGG (left panel, up-regulated DEGs) and GSEA (right panel, *P* < 0.05) enrichment analysis between CD52^high^ and CD52^low^ MNB tumors. **G** Activated CD8^+^ T cells were exposed to CD52-Fc treatment or a full-length overexpression (AdCD52) procedure and then subjected to an apoptotic assay after coculture with NB cells for 48 h. Fc and AdmNG (ADV-mNeonGreen) are controls for CD52-Fc and AdCD52, respectively. Statistics analyses under cell lines cocultured were shown in the right panel (top:SK-N-AS,bottom:SK-N-SH). **H** ROS accumulation in CD8^+^ T cells treated as indicated was measured by DCFH-DA assay. Ctrl stands for the average of undistinguishable controls of Fc and AdmNG. **I** RT-PCR analysis of mRNA levels, including the apoptotic-related genes *BAX*, *MCL-1*, *and Bcl-2*, as well as immune checkpoint molecules (*PDCD1*, *CTLA4*, and *HAVCR2*), in AdCD52 or AdmNG CD8^+^ T cells. ^*^P & ^#^*P* < 0.05, ^**^P < 0.01, ^***^P & ^###^*P* < 0.001, ^****^P < 0.0001. The data in G-I represent the mean ± SD (*n* = 3)

Correlation analysis integrating immunofluorescence and transcription levels of MNB highlighted the most positive connection between CD52 and activated CD8^+^ T cells (Fig. 2A, B). We took advantage of RNA microarray data from 498 NB samples (GSE49710) to validate the prognostic preference of CD52 expression on CD8^+^ T cells. Consistently, *CD52* was more correlated with *CD8A* than *CD4* (R = 0.77 *v.s.* 0.58 in the MNB dataset and R = 0.72 *v.s.* 0.48 in the GSE49710 dataset) (Fig. 2C, Fig. S2A), indicating CD52 was functional weighted on CD8^+^ T cells in NB.

Notwithstanding that the cohort with higher *CD8A* and *CD52* expression exhibited better event-free survival (EFS) and overall survival (OS) probabilities, independently (Fig. S2B, S2D), both of which were less expressed separately in NB patients with high-risk, MYCN amplification, progression, and advanced stage (Fig. S2C, E). When splitting the matrix into two subcohorts according to the median level of *CD8A* expression (CD8A^high^ and CD8A^low^), we observed a relatively poor prognosis of *CD52* in the CD8A^high^ subcohort (Fig. 2D). Moreover, *CD52* was mostly expressed in advanced stage, progressed, and high-risk NB patients in the CD8A^high^ subcohort, in contrast to the total GSE49710 samples and CD8A^low^ subset (Fig. 2E). We further divided the MNB matrix into CD52^high^ and CD52^low^ subsets and observed CD8^+^ T cells were more infiltrated in the CD52^high^ subset than in the CD52^low^ subset (Fig. S2F). Among these, checkpoint molecules and activated CD4^+^ T cells were also predominantly expressed (Fig. S2F). Functionally, DEGs were also highlighted by the primary immunodeficiency pathway in the CD52^high^ cohort (Fig. 2F, Fig. S2G). These findings hypothesize that CD8^+^ T cells with the highest CD52 expression were functionally inhibited and responsible for poor survival in CD8^+^ T-infiltrated NB.

### CD52-overexpressed CD8^+^ T cells undergoes apoptotic events in the presence of NB cells

The cross-linking effect of CD52 on T cells activates intracellular apoptotic signals through tyrosine phosphorylated proteins [46]. To determine whether soluble CD52 (CD52-Fc) or endogenous CD52 expression anchors NB-inflamed CD8^+^ T cells with suppressor activity, CD8^+^ T cells were effectively subjected to full-length CD52 (AdCD52) transfection or CD52-Fc incubation (Fig. S2H-J). A reduction in cell living rate was observed in CD8^+^ T cells treated with AdCD52 and CD52-Fc (Fig. S2K), with this effect being more obvious when cocultured with NB cell lines (Fig. 2G). Overexpression of CD52 was also able to up-regulate ROS levels (Fig. 2H), the expression of apoptotic regulatory gene *BAX*, and the mRNA levels of immune checkpoint molecules (*PDCD1*, *CTLA4*, *HAVCR2*), while down-regulating anti-apoptotic *MCL-1* and *Bcl-2* levels in CD8^+^ T cells (Fig. 2G), illustrating the significant role of CD52 in triggering suppression signals in NB-activated CD8^+^ T cells.

### The dysfunction of MNB-infiltrated CD8^+^ T cells induced by EDF1 in NB cells are responsible for the unfavorable prognosis

**Fig. 3:**
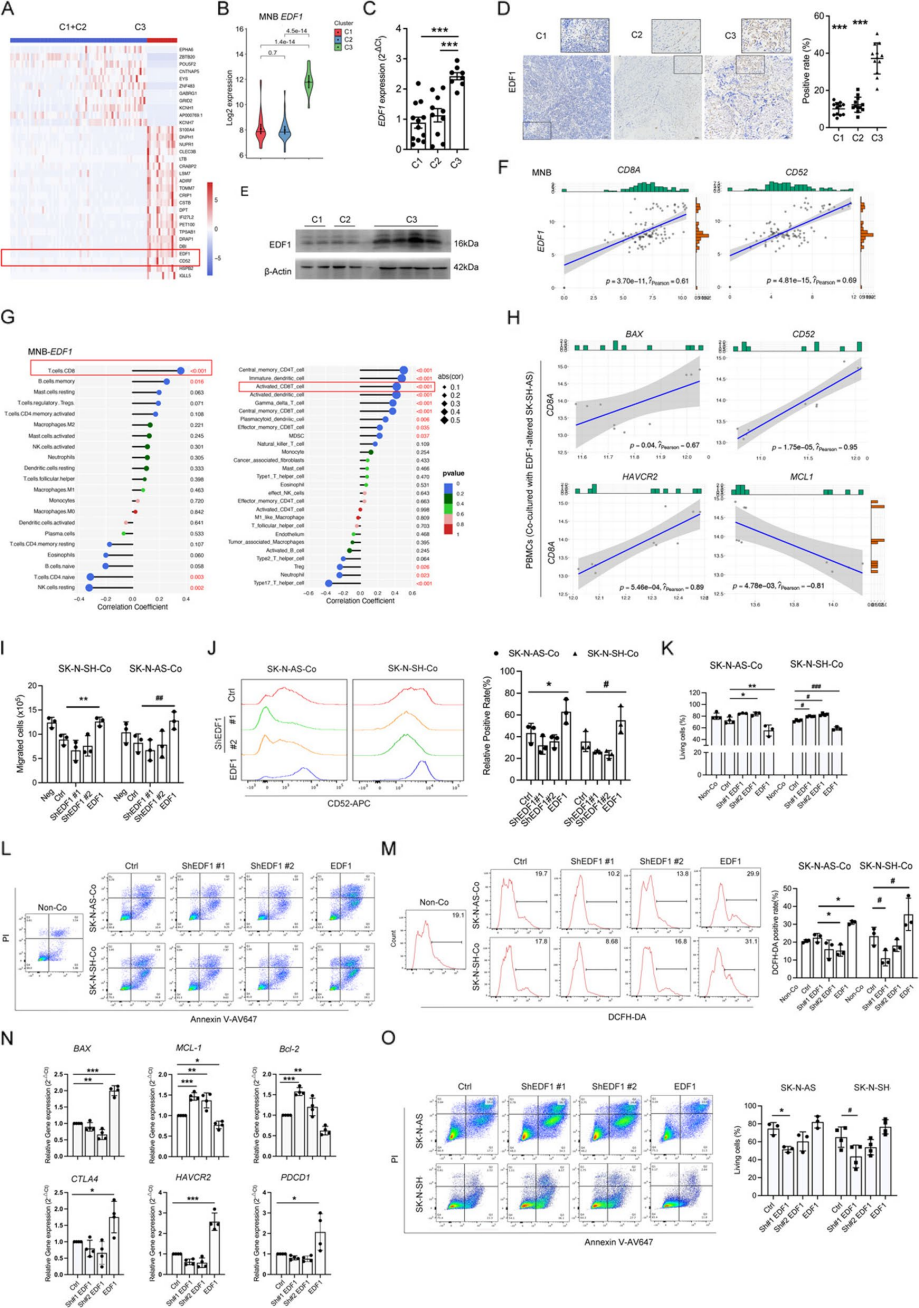
EDF1 in NB cells accelerates the chemotaxis of CD8^+^ T cells and functions as an upstream regulator of CD52-mediated CD8^+^ T cell suppression. **A** Heatmap for DEGs between C1 + C2 and C3 (log2fold change > 3 & log2fold change < −5 & *P* < 0.01).** B** Log2 expression of *EDF1* in the transcriptional matrix across the three clusters. **C, D, E** qRT-PCR, IHC and immunoblotting analyses of EDF1 expression across the three clusters. *n* = 4 in each group. The average positive ratio from 3–5 fields was counted by symbol and color. The scale bars represent 50 μm. **F** Correlation analyses between *EDF1* and *CD8A*, as well as *CD52* in MNB tumors.** G** Correlation analyses between *EDF1* and immune components in MNB tumors. The size of the dots represents the correlated strength, and the color of the dots represents the accordant *P* value. **H** Correlation analyses between *CD8A* and *CD52, HAVCR2, BAX*, and *MCL1* in the RNA-seq matrix of PBMCs cocultured with EDF1-altered NB cells. **I** The chemotaxis of CD8^+^T cells induced by CM (conditioned medium) from EDF1-altered NB cells was analyzed by counting migrated cells in the bottom chamber of transwell inserts.** J** Left panel, representative flow cytometry histograms of CD52 level in CD8^+^ T cells cocultured with EDF1-altered NB cells. The percentages are shown and analyzed in the right panel. **K-N** Apoptosis, ROS level and qRT-PCR analyses in CD8^+^ T cells cocultured with EDF1-altered NB cells. The ratio of live cells and DCF-positive cells are shown and analyzed in (**L**) and **(M**, Right), separately. **O** Representative apoptotic plots of NB cells exposed to supernatant from the EDF1-altered coculture system. The ratio of living cells is shown in the right panel. Ctrl in I-O stands for the average of undistinguishable controls of scrambled sequence (for Sh-EDF1) and empty vector (for EDF1 overexpression). The data in I-K, M–O represent the mean ± SD (*n* = 3). ^*^P & ^#^*P* < 0.05, ^**^P & ^##^*P* < 0.01, ^***^P &.^###^*P* < 0.001

To identify potential upstream factors contributing to elevated CD52 levels in CD8^+^ T cells, C1 and C2 were merged as a set with lower CD8^+^ T cell infiltration compared with C3. Notably, EDF1 was mostly up-regulated in C3, as evidenced by its favorable log2 fold change and *P* value (Fig. 3A, B). The highest mRNA and protein levels of EDF1 in C3 were further validated (Fig. 3C-E). We observed that *EDF1* level was highly correlated with *CD8A* (R = 0.61) and *CD52* (R = 0.69) levels, as well as CD8^+^ T cell number (Fig. 3F, G). suggesting EDF1 in NB cells engaged the function of CD8^+^T cells. Although EDF1 expression hardly exhibited statistical differences in the risk and stage of MNB tumors (Fig. S3A, S3B), we observed a significant up-regulation of *EDF1* levels in NB patients with high-risk, advanced stage, MYCN amplification, and progression (Fig. S3D), accompanied by lower OS and EFS (GSE49710) (Fig. S3C). Thus, we hypothesize that overexpressed-EDF1 in NB cells orchestrates CD52 expression in CD8^+^ T cells, inducing unfavorable outcomes.

### High EDF1 expression in NB cells accelerates the chemotaxis of CD8^+^ Tcells and acts as an upstream regulator of CD52-mediated CD8^+^ T cell suppression

Cellular EDF1 expression was detected in both MYCN-amplified and MYCN-nonamplified NB cell lines (GSE19274) without any discernible preference (Fig. S3E). We selected five NB cell lines (SK-N-SH, SK-N-BE2, SK-N-AS, SH-SY5Y, and IMR32) for further analysis, and observed the mRNA and protein levels of EDF1 were abundant in SK-N-AS and SK-N-SH cells (Fig. S3F). To investigate the impact of EDF1 on the behavior of NB cells and NB-associated CD8^+^ T cells, we engineered knockdown and full-length overexpression plasmids of EDF1 and validated its endogenous expression (Fig. S3G, H). Alterations in EDF1 expression in NB cells significantly controlled their migration rather than proliferation (Fig. S3 I-K). We then cocultured EDF1-altered NB cells with PBMCs from NB patients. The RNA-seq matrix mixed with NB-activated immune cells also highlighted that *CD8A* expression was positively correlated with that of *CD52, HAVCR2, and BAX*, but negatively correlated with *MCL1* level (Fig. 3H). Consequently, we focused on the re-education of CD8^+^ T cells by EDF1-altered NB cells.

The chemotaxis of CD8^+^ T cells, rather than their proliferation, was also dominated by the CM from EDF1-altered NB cells (Fig. 3I, Fig. S3L). This parallel behavior in NB cells led us to hypothesize that the enhanced migratory ability of both NB and CD8^+^ T cells might be driven by some common soluble seducers induced by EDF1. Additionally, CD52 expression and CD8^+^ T cell apoptosis were both in accordance with EDF1 levels in NB cells in the coculture system (Fig. 3J-L). The levels of suppressive genes (*BAX*, *PDCD1*, *CTLA4*, and *HAVCR2*) and ROS showed similar tendencies, while the expression of anti-apoptotic genes (*MCL-1 and Bcl-2*) were reversely regulated (Fig. 3M, N). Furthermore, supernatant from the EDF1 knockdown cocultured system also induced more apoptotic events in NB cells, which was dampened by elevated EDF1 levels (Fig. 3O). The abovementioned data illustrates that high level of endogenous EDF1 in NB cells promotes its migration, induces CD8^+^T cell chemotaxis, and serves as an up-regulator of CD52-mediated CD8^+^T cell suppression.

### Lipid metabolism and identification in MNB by lipidomic analysis

**Fig. 4:**
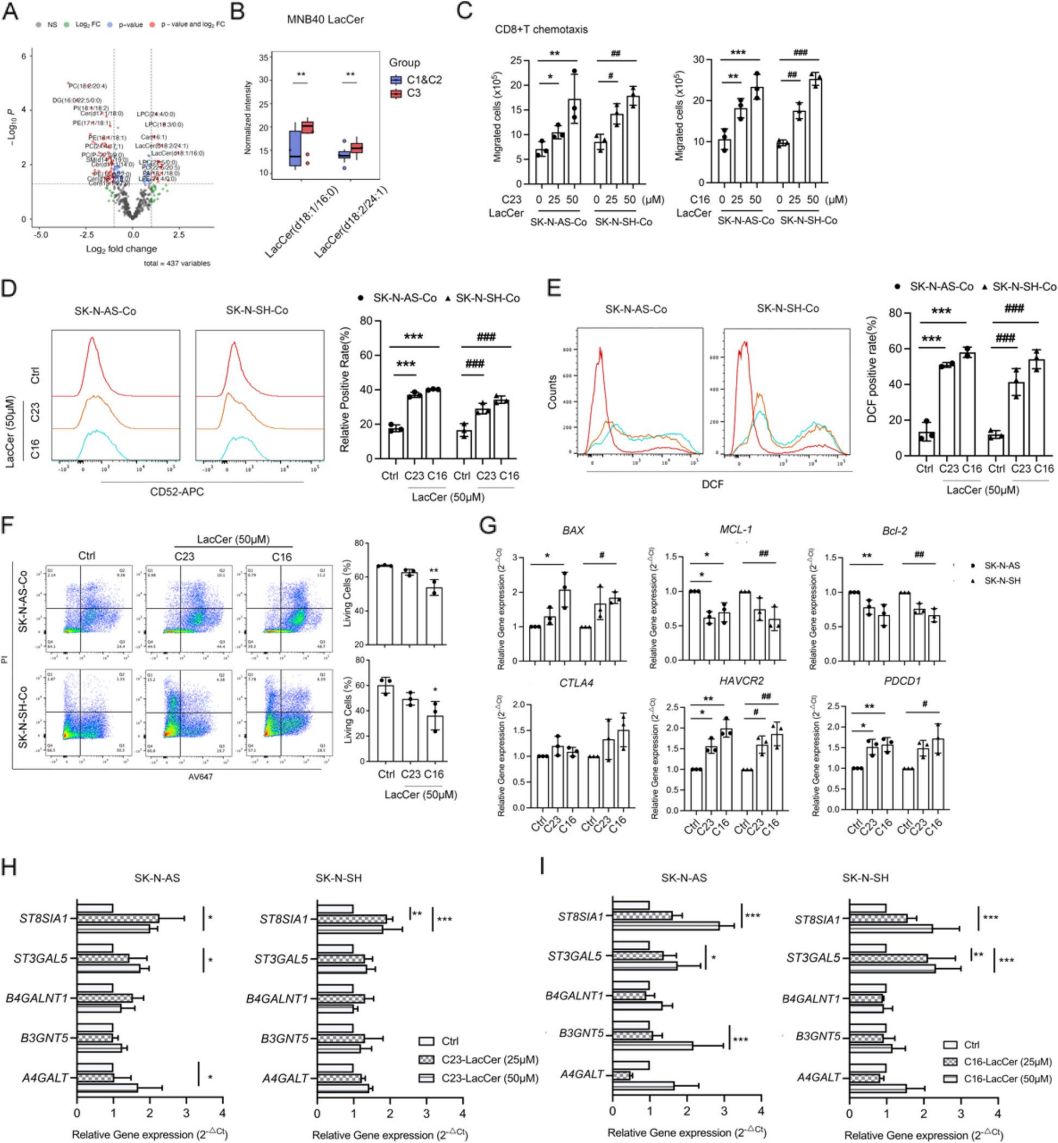
LacCers in MNB tumors possess pro-migration activity and mediates CD8^+^ T cell suppression. **A** Volcano plot of differential lipidomic metabolites (C1 + C2 *v.s.* C3, |log2fold change|> 1 and *P* value < 0.05) in MNB tumors (*n* = 42), according to mass spectrometry analysis. Normalized intensities of LacCer (d18:1/16:0) and LacCer (d18:2/24:1) were statically analyzed in (**B**). ^**^P < 0.01. **C** The chemotaxis of CD8^+^ T cells induced by exogeneous C23 and C16 LacCer-treated NB cells was analyzed by counting migrated cells in the bottom chamber of transwell inserts. **D**, **E**, **F** Left panel, representative flow cytometry histograms of CD52 (**D**) and ROS levels (**E**) and plots of apoptosis (**F**) in CD8^+^ T cells cocultured with C23 and C16 LacCer-treated NB cells. The percentages of positive staining are shown in the corresponding right panel.** G** qRT-PCR analyses of dysfunctional-regulated genes in CD8^+^ T cells cocultured with C23 and C16 LacCer-treated NB cells. **H**, **I**, qRT-PCR analyses of gangliosides synthases in C23 and C16 LacCer-treated NB cells. The data above represent the mean ± SD (*n* = 3). ^*^P < 0.05, ^#^*P* < 0.05, ^**^P & ^##^*P* < 0.01, ^***^P &.^###^*P* < 0.001

EDF1 mainly functions as a transcriptional coactivator for *PPARs* expression and is necessary for regulating lipid synthesis and metabolism [47]. we performed lipidomic analysis in the three clusters (Fig. S4A). A total of 70 differential lipids emerged when comparing C1 + C2 with C3 (|log2fold change|> 1 and *P* value < 0.05) (Fig. 4A), among which LacCers (d18:1/16:0 and d18:2/24:1) were enriched in C3 (Fig. 4B). We found both exogeneous C23 and C16 LacCer showed similar tendencies with EDF1 overexpression. Treatment with LacCers also dose-dependently increased the migration ability of NB cells (Fig. S4C, D). LacCer-treated NB cells accelerated the chemotaxis, CD52 expression, and apoptotic-related events in cocultured CD8^+^ T cells (Fig. 4C-G), while the change of proliferative activities were not apparent (Fig. S4E).

LacCer is predominantly generated by LacCer synthases [14]. We analyzed the RNA-seq data combined with lipidomic data to seek the crosstalk among *EDF1*, *CD52*, and lactose synthases of GSLs. A positive correlation of *ST3GAL5* with *EDF1* (R = 0.52) and *CD52* (R = 0.33) was respectively observed (Fig. S4F), while others showed trivial or opposite values. Additionally, both *EDF1* and *CD52* also showed positive correlation with *CERS4* and *CERS5*, two family members in LacCer synthases (Fig. S4F). Interestingly, the expression of a- and b-series synthases for gangliosides was increased in NB cells exposed to C23 and C16 LacCers, especially *ST3GAL5* and *ST8SIA1* (Fig. 4I). Considering that gangliosides are central to many cellular functions and promote cell migration [16, 48], we hypothesize that LacCers synthases or downstream gangliosides act as EDF1-mediated factors to trigger CD8^+^ T cell suppression.

### EDF1 engages LacCer-ganglioside metabolism

**Fig. 5:**
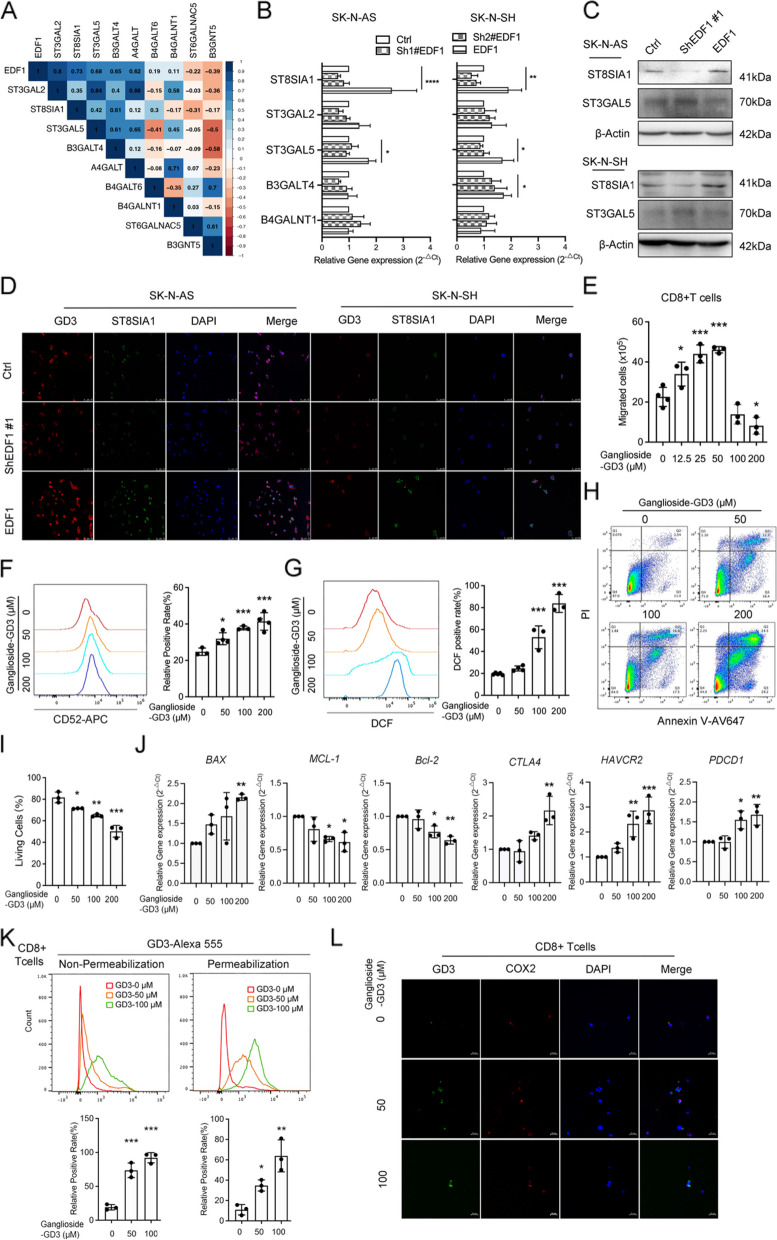
EDF1 accelerates GD3 secretion of NB cells and inhibit the functions of CD8^+^T cells by recruiting to mitochondrial microdomain. **A** Correlation plots among EDF1 and synthases for gangliosides in an RNA sequencing matrix involving Ctrl, EDF1-overexpressing and EDF1-knockdown SK-N-AS cells. **B** qRT‒PCR analyses of synthases for gangliosides in EDF1-altered NB cells. **C** Immunoblotting analyses of ST8SIA1 and ST3GAL5 in EDF1-altered NB cells. β-Actin was used as a loading control for immunoblotting. Ctrl in A-C stands for the average of undistinguishable controls of scrambled sequence (for Sh-EDF1) and empty vector (for EDF1 overexpression).** D** Representative immunofluorescence staining images of ST8SIA1 (green) and GD3 (red) in EDF1-altered NB cells. DAPI (blue) was used to stain nuclei. **E** The chemotaxis of CD8^+^T cells induced by EDF1-altered NB cells was analyzed by counting migrated cells in the bottom chamber of transwell inserts.** F**, **G** Representative flow cytometry histograms of CD52 (**F**) and ROS levels (**G**), in CD8^+^T cells exposed to exogenous GD3. The percentages of positive staining were statistically analyzed. (**H-J**) Representative flow cytometry plots of apoptosis (**H**) and mRNA levels of apoptosis-related genes in CD8^+^T cells exposed to exogenous GD3. The percentages of living cells were statistically analyzed in (**I**). (**K**) Representative flow cytometry histograms of GD3 in CD8^+^T cells with or without permeabilization exposing to exogenous GD3. The percentages of positive staining were statistically analyzed. (**L**) Representative immunofluorescence staining images of COX2 (green, mitochondrial inner membrane) and GD3 (red) treated as indicated. DAPI (blue) was used to stain nuclei. The scale bar represents 20 µm. The data in B-L represent the mean ± SD (*n* = 3). ^*^P < 0.05, ^**^P < 0.01, ^***^P < 0.001

We subsequently investigated the transcriptomic impact of EDF1 on LacCer metabolism (Fig. S5A, B). EDF1 was positively correlated with pathways involved in cell migration, calcium signaling, and GSL biosynthesis (Fig. 5SC, D), as well as with the expression of ganglioside and LacCer synthases (Fig. 5A, Fig. 5SE). LacCer can be produced from the degradation of ganglioside-GM3 or -GD3 by NEU3 [49], suggesting an increased presence of gangliosides in EDF1-overexpressing NB cells. Combined with the characteristics of LacCer-treated NB cells, *ST8SIA1* was identified as a downstream target of EDF1 (Fig. 5B, C).

### EDF1-elevated GD3 content in NB cells upregulates CD52 in CD8^+^ T cells and inhibits its activity

Ganglioside-protein interactions in cancer cells are capable of suppressing T-cell and B-cell cytotoxicity [21]. ST8SIA1 is responsible for GD3 synthesis from GM3, contributing to the invasive behavior of cancer cells [50]. Our findings indicated that GD3 accumulation occurred in EDF1-overexpressed NB cells, with a reduction in intensity upon EDF1 knockdown (Fig. 5D). Furthermore, treatment with GD3 disodium salt dose-dependently increased chemotaxis (less than 50 μM), CD52 expression, ROS levels, and apoptosis-related events in CD8^+^ T cells (Fig. 5E-J), indicating that the elevated GD3 content induced by EDF1 in NB cells may primarily account for the suppression of CD8^+^ T cell activity.

### GD3 secreted by NB cells can be recruited to the mitochondrial membrane of CD8^+^ T cells and contributes to its functional arrest

We then traced GD3 in CD8^+^ T cells after exposure to exogenous GD3 treatments, to characterize the effect of its trafficking on the fate of CD8^+^ T cells. Markedly, GD3 levels increased in a dose-dependent manner, especially in permeabilized CD8^+^ T cells (Fig. 5K). A part of GD3 translocated from the cell membrane to cellular microdomains, where it co-localized with COX2, a component of cytochrome c oxidase in the inner mitochondrial membrane (Fig. 5L), indicating that the apoptotic events of CD8^+^ T cells are mitochondria-related and triggered by intracytoplasmic GD3.

### EDF1/RelA interaction accelerates *ST8SIA1* expression in NB cells

**Fig. 6:**
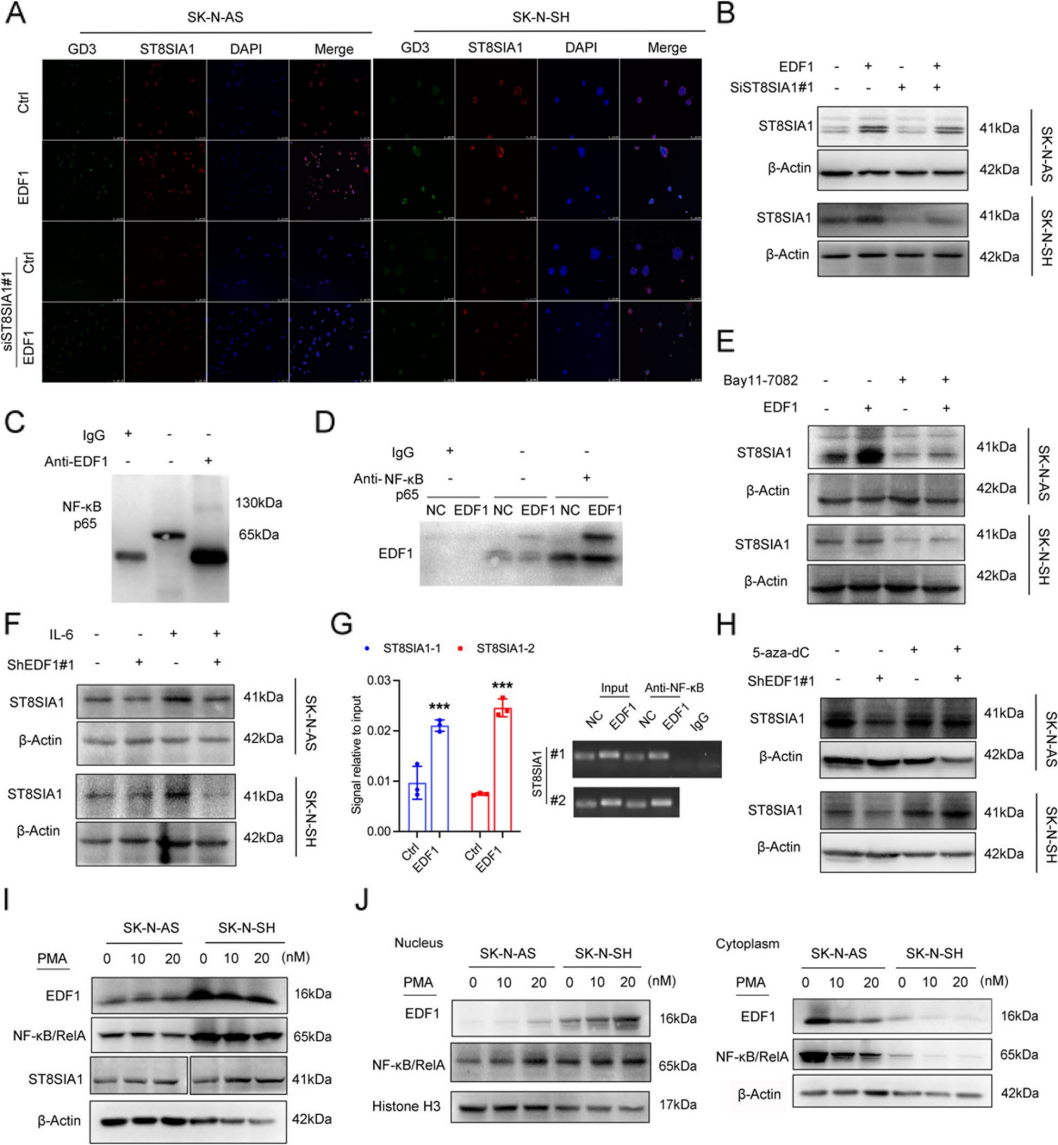
EDF1 accelerates NF-κB/RelA binding to the promoter of *ST8SIA1* to avoid methylation.** A** Representative immunofluorescence staining images of ST8SIA1 (green) and GD3 (red) treated as indicated. DAPI (blue) was used to stain nuclei. **B**, **E**, **F**, **H** Immunoblot analysis of ST8SIA1 expression in cells treated as indicated. β-Actin was used as a loading control. **C**, **D** Coimmunoprecipitation (Co-IP) assays were performed to verify the interaction between NF-κB/RelA and EDF1. **G** ChIP-PCR assays showed that 54 ~ −63 bp of the ST8SIA1 promoter contains a consensus NF-κB/RelA binding region.** I**, **J** Immunoblot analysis of NF-κB/RelA, ST8SIA1 and EDF1 expression in the cytoplasm and nucleus of NB cells, as well as their total levels after treatment with PMA. β-Actin was used as a loading control in both the cell cytoplasm and total cell lysates, and histone H3 was used as a loading control separately in the cell nucleus. The data in G represent the mean ± SD (*n* = 3). ^***^P < 0.001

Cellular *ST8SIA1* was equally expressed among NB cell lines (Fig. S6A). Utilizing siRNAs targeting *ST8SIA1* in NB cells (Fig. S6B), we observed that *ST8SIA1* knockdown significantly decreased GD3 and ST8SIA1 levels in both Ctrl and EDF1-overexpressed NB cells (Fig. 6A, B). Further, Ctrl- and si#ST8SIA1-NB cells were both exposed to cisplatin (CDDP), one of the most common chemical drugs used to treat NB, or following by coculturing with CD8^+^ T cells. As expected, si#ST8SIA1-NB cell was more sensitive to CDDP, while coculturing with CD8^+^T cells (Fig. S6C, D), indicating that GD3 inhibition accelerates the response of NB cells to chemotherapies. Conversely, exogenous C23 and C16 LacCers hardly elevated GD3 content in EDF1-knockdown NB cells (Fig. S6E), indicating that EDF1 predominantly regulates ST8SIA1 level, which is critical for LacCer-induced GD3 content.

EDF1 is located both in the cytosol and nucleus of endothelial cells [51]. Furthermore, the nuclear translocation of EDF1 acts as a transcriptional coactivator [52], to form a transcription initiation complex. According to the identified peptides from mass spectrometry, RelA showed binding potential with EDF1 in the NC group, which was twofold higher in EDF1-overexpressed NB cells. As a critical subunit of the transcription factor NF-κB, RelA can trigger a series of inflammatory and immune responses via a canonical pathway and is also involved in *ST8SIA1* transcriptional activation [53]. We identified a dimeric Rel/NF-κB protein band at nearly 130 kDa, which was immunoprecipitated by EDF1, but obscured by a monomeric isoform (65 kDa) in the input lysates (Fig. S6F, Fig. 6C). Additionally, EDF1 was immunoprecipitated by an anti-RelA antibody in both NC and EDF1-overexpressed NB cells (Fig. 6D), suggesting that the dimeric Rel/NF-κB is responsible for DNA binding and the subsequent transcriptional activation of *ST8SIA1*.

We took advantage of IL6 and Bay11-7082, as inducers and inhibitors of NF-κB/RelA translocation, respectively. It exhibited that up- or down-regulated EDF1 seldom altered the distribution of NF-κB/RelA and EDF1 (Fig. S6G, H). However, both ST8SIA1 levels at baseline and elevated by EDF1-overexpression were blunted by Bay11-7082 (Fig. 6E). Moreover, the upregulation of ST8SIA1 stimulated by IL6 was scarcely detectable when EDF1 expression was knocked down (Fig. 6F). Our results indicate that nuclear EDF1 acts as a transcriptional coactivator through binding with NF-κB/RelA and promotes ST8SIA1 transcription in NB cells.

### EDF1/RelA complex accelerates *ST8SIA1 *transcription by blocking its methylation after binding to *ST8SIA1* promoter

According to the prediction from JASPAR database (version 2020) [54], NF-κB/RelA exhibited higher binding affinity at the *ST8SIA1* promoter (−54 ~ −63 bp) (Fig. S6I). The binding sites were further confirmed by ChIP assays and ChIP-PCR (Fig. 6G), with two pairs of primer targeting −54 ~ −63 bp of the *ST8SIA1* promoter detailed in Table S5. Notably, the upstream 788 bp region (ATG) of the transcriptin start site is essential for *ST8SIA1* transcription [55] and is characterized by a CpG island inclined to be methylated, which hinders the binding of NF-κB/RelA. To determine whether *ST8SIA1* expression was inversely correlated with methylation, we used 5-aza-2’-deoxycytidine (5-aza-dC) to demethylate *ST8SIA1* promoter, which restored *ST8SIA1* expression in EDF1 knockdown NB cells (Fig. 6H). Consequently, EDF1 functions as a coactivator in NF-κB/RelA/EDF1 complex that binds to *ST8SIA1* promoter, preventing its methylation and subsequently promoting its transcription in NB cells.

### Dissociation of cytosolic EDF-1/CaM facilitates nuclear EDF1-mediated *ST8SIA1* transcription

Calmodulin (CaM) is a Ca^2+^ receptor that binds with EDF1 in the cytosol, of which the binding activity is sensed by the modification state of EDF1 and the concentration of intracellular free Ca^2+52^. Upon stimulation with phorbol 12-myristate 13-acetate (PMA), a PKCα activator, which enhances intracellular Ca^2+^ signaling [56], the overall levels of EDF1 and NF-κB p65/RelA, as well as the translocation of NF-κB p65/RelA remained stable. However, EDF1 in the cytosol was dose-dependently reduced, accompanied by increased EDF1 nuclear accumulation and total ST8SIA1 expression (Fig. 6I, J). This finding suggests that the EDF1/CaM interaction in NB cells is tightly regulated by Ca^2+^ level, and the dissociation of EDF1 from cytosolic CaM facilitates nuclear EDF1-mediated DNA binding with NF-κB p65/RelA, leading to the subsequent transcriptional activation of *ST8SIA1*.

### EDF1 induces NB tumor progression by deactivating CD8^+^ T cells in vivo

**Fig. 7:**
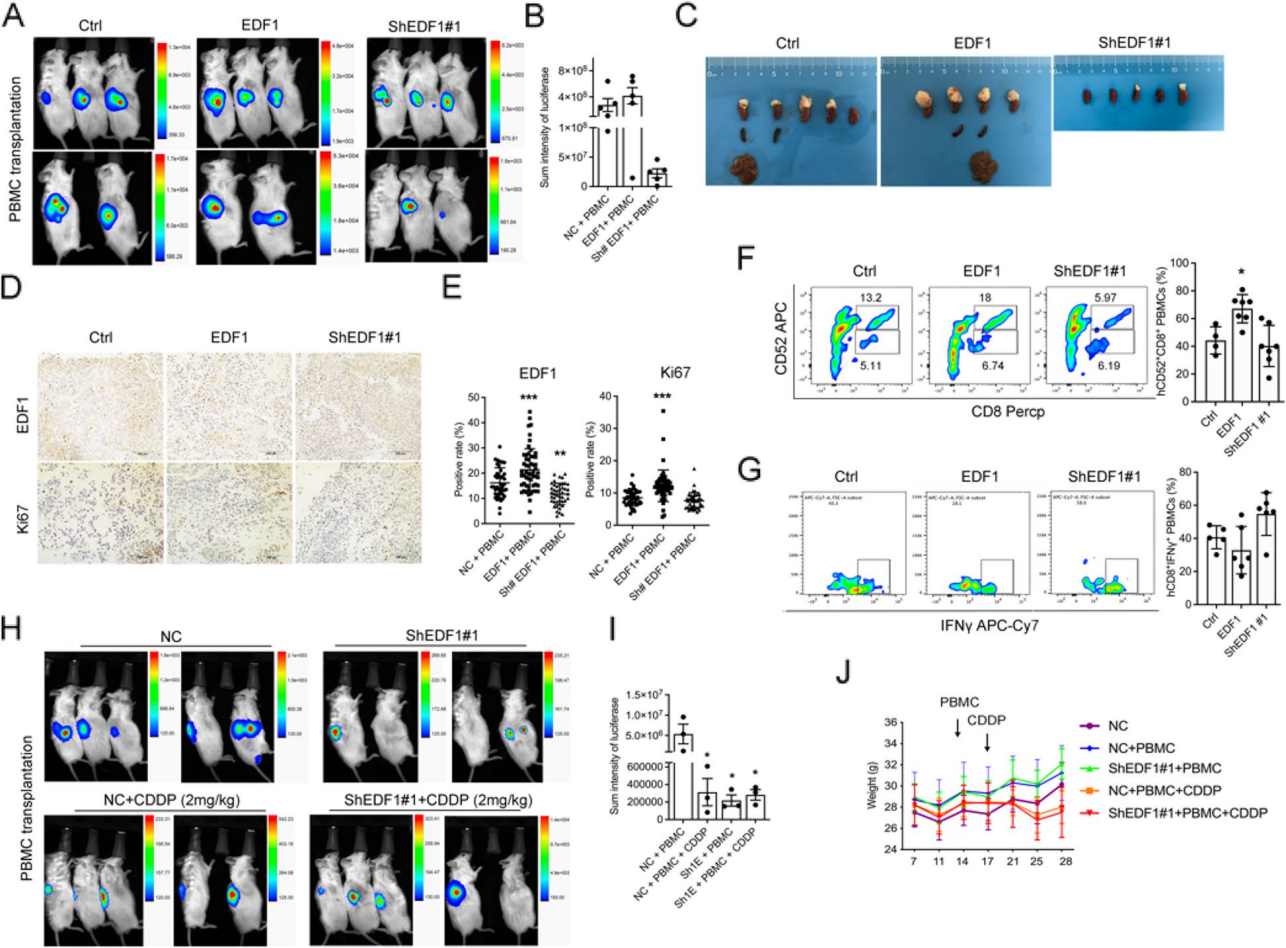
EDF1 promotes NB tumor progress by deactivating CD8^+^ T cells in vivo***. *A-B** Orthotopic NB models were generated by injecting EDF1-altered SK-N-SH cells into the left adrenal gland of B-NDG mice, followed by transplanting human PBMCs. Representative luciferase images are shown in (**A**), and light emission was captured and quantified by bioluminescence (**B**). The data are presented as the mean ± SD (*n* = 5). ^*^p < 0.05; ^**^p < 0.01. **C** Visual examination of primary and metastatic sites in mice of each group. **D**, **E** Representative pictures of Ki67 and EDF1 staining in fixed primary pieces from each group. The scale bars represent 100 µm. The average positive ratio from 3–5 fields was analyzed by symbol and color and is shown (**E**). **F**, **G** Representative flow cytometric plots of human CD45^+^ lymphocytes isolated from tumors in each group and further staining with human CD8, CD52 and IFNγ. **H-J** Humanized orthotopic NB models from Ctrl and EDF1-knockdown SK-N-SH cells were both subjected to CDDP (1 mg/kg). Representative luciferase images are shown in (**H**), light emission (**I**), as well as body weight (**J**) were quantified and mornitored separately. The data are presented as the mean ± SD (*n* = 5). ^*^P < 0.05; ^**^P < 0.01

An orthotopic NB model was established by injecting SK-N-SH cells with altered EDF1expression [24]. After hPBMC transplantation, no significant difference in survival rate was found (1/10 in NC, 2/10 in EDF1 group were died). However, EDF1 overexpression enlarged tumor size compared to Ctrl, while down-regulation of EDF1 inhibited tumor growth and metastasis (spleen and liver) (Fig. 7A-C, Fig. S7A), as validated by EDF1 and Ki67 staining (Fig. 7D, E). Meanwhile, CD45^+^ hPBMCs were monitored and found to stably existed in the blood and tumors of mice (Fig. S7B, C), with the highest infiltration observed in tumors overexpressing EDF1. Additionally, a higher ratio of CD52^+^ to CD52^−^ cells within CD8^+^ CD45^+^ T cells was observed in tumors, rather than blood, in the EDF1-overexpressing group (Fig. 7F, Fig. S7D). This was accompanied by a relatively lower presence of IFNγ^+^ CD8^+^ T cells (Fig. 7G), indicating that EDF1-promoted tumorigenesis was assisted through the inhibition of CD8^+^ T cells in vivo. Furthermore, although EDF1 knocked down NB cells seemed more sensitive to cisplatin (CDDP), the reduction in tumor size.

was similar to that of CDDP treatment (Fig. 7H, I). Additionally, the tumor-inhibiting effect by knocking down EDF1 was rarely caused potential toxic effects, such as body weight loss, comparing with CDDP treatment (Fig. 7J). These results reveal a potential alternative therapy for NB treatment to mitigate the toxicity of CDDP.

## Discussion

Our study employs the NMF algorithm of RNA-sequencing data to classify MNB samples and elaborates the characteristics of immune elements educated by MNB. First, we noticed the identified three clusters were not discrete by the customary classification, indicating high heterogenicity of NB. Although previous study had clustered NB into Hi-MYCN, neuronal, immunogenic and metabolic subtypes basing on NB transcription profiles [2]. We acquired ssGSEA scores of the four NB subtypes in our dataset, and found immunogenic and metabolic markers were both in low levels among the three clusters, while Hi-MYCN-NB was obvious expressed in C3, which owns the most high-risk samples. C1 exerted infertile immune cell infiltration but enriched in neuronal markers (data not shown), raising the possibility that neuronal-NB was negatively associated with tumor burden.

During tumor progression, only a small fraction of TILs is activated to recognize autologous tumor cells, most of them exist in a memory state over the long term as “bystander” cells [57]. Moreover, the OS of NB is partly attributed to the ‘ineffective’ presence of TILs, defined as an exhausted state with reduced production of effectory cytokines [58]. In our study, we demonstrated the prognosis of MNB was largely depends on the status of intratumor CD8^+^T cells, and verified though in vitro and in vivo experiments. Therefore, reeducating CD8^+^T cells may be crucial for generating an effective immune response against MNB tumors.

Among the highly expressed genes in C3, we focused on CD52 due to its specific suppressive activity in T cells. The mature form of human CD52 consists of 12 amino acids and is the target of the Campath-1H antibody (Alemtuzumab) [45]. Beyond its complement-mediated elimination and antibody-dependent cell-mediated cytotoxicity (ADCC) effects [59], activated CD4^+^ CD52^high^ T cells also exhibit suppressor activity through the release of soluble CD52, which acts as a ligand for Siglec-10 [60]. The binding of CD52 to Siglec-10 leads to decreased phosphorylation of the T-cell receptor-associated proteins Lck and ZAP-70. Soluble CD52 not only suppresses the secretion of inflammatory cytokines by innate immune cells, but also triggers intrinsic apoptotic cell death by depleting MCL-1. Analysis of the transcriptional profiles from the MNB and GEO datasets revealed a distinctive association between poor prognosis and CD52 expression in the CD8A^high^ subcohort. Together with cellular data that highlighted increased apoptotic events in CD8^+^ T cells induced by CD52, we proved CD52 as a typical immunosuppressor of CD8^+^T cell activation in NB patients.

In NB cells, EDF1 was identified as an upstream candidate responsible for chemotaxis, elevated CD52 level, and apoptotic events in CD8^+^T cells. Furthermore, the transplanted hPBMC-NB model verified that the inhibition of suppressive factors and reactivation of CD8^+^T cells could be achieved through knocking down EDF1 in NB cells. EDF1 knockdown proved to be more advantageous than CDDP in NB therapy with trivial toxic effects. Further improved in vivo assays are essential to support our hypothesis. EDF1 is ubiquitously expressed in both cytosol and nucleus. In cytosol, it serves as a regulator of calmodulin availability [52, 61, 62], a Ca^2+^-binding protein that modulates several calcium-regulated enzymes. For instance, EDF1 can induce nitric oxide (NO) release by targeting endothelial NO synthase (eNOS) [63]. During translational distress, EDF-1 recruits translational repressors to prevent the generation of new ribosomes translated by defective mRNAs and coordinates pathways mediated by cellular ribosome collisions [64]. In addition, the dissociation of cytosolic EDF1 from CaM in the presence of PMA enhances its nuclear accumulation and transcriptional co-factor activity [52, 61]. As a transcriptional co-factor, EDF1 has been reported to modulate lipid metabolism by binding to lipid-associated receptors, such as liver receptor homolog 1 (LRH-1), liver X receptor (LXR)-alpha, and peroxisome proliferator-activated receptor (PPAR)-gamma [47, 51, 65]. Here, we uncovered the transcriptional activation of EDF1 on *ST8SIA1* through targeting dimeric Rel/NF-κB, leading to the accumulation of LacCers and subsequent synthesis of ganglioside-GD3. Our findings highlight the potential for a combinatorial approach to improve the efficacy of current NB therapies.

Most gangliosides are synthesized by specific glycosyltransferases. In our study, GD3 synthetase (ST8SIA1) was more sensitive to EDF1 alteration than GD2 synthetase (ST3GAL5). ST8SIA1 is essential for the acquisition of invasive and migratory behaviors in tumors [50]. Additionally, we confirmed the suppressive activities of ST8SIA1-synthesized GD3 on CD8^+^ T cells. Previous study revealed that pro-apoptotic signaling in T cells is influenced by the intracytoplasmic transport of glycosphingolipids via microtubules [66], with mitochondria identified as a potential target. Consistently, we uncovered exogenous GD3 was anchored in both the cell membrane and mitochondria of CD8^+^T cells. We hypothesized that GD3 could migrate from the cell plasma membrane to the mitochondria by binding to polymeric microtubules in CD8^+^ T cells. Specific works are need to illustrate the crosstalk between CD52-mediated apoptosis in CD8^+^ T cells and the translocation of mitochondria-anchored GD3. In this scenario, the LacCer-GD3 axis acts as a key mediator between EDF1 in NB cells and CD52 expression in CD8^+^ T cells, playing a crucial role in facilitating the immune escape of NB cells. This finding holds potential significance in the field of immunotherapy, as alterations in ganglioside composition may be useful in overcoming the exhaustion and enhancing the activation of CD8^+^ T cells during anti-tumor treatments.

## Conclusion

In summary, the integration of transcriptomic and lipidomic data in MNB samples has provided a better understanding of the interaction between lacCer metabolites and TME status. Our study reveals that EDF1-induced GD3 synthesis through the RelA/ST8SIA1 axis in NB cells predominantly contribute to its suppressive effect on CD8^+^ T cells, highlighting its potential as an immunotherapeutic regulator. Nevertheless, challenges remain in sustaining and prolonging the cytotoxic activities of effector cells. Hence, further investigation is required to fully elucidate the mechanism by which the GD3/CD52 axis inactivates CD8^+^ T cells. Overall, regulating NB-associated GD3 level is beneficial for maintaining CD8^+^T cell activity and tuning the depth of responses to current NB therapies.

## Supplementary Information

Supplementary Material 1.

Supplementary Material 2.

Supplementary Material 3.

Supplementary Material 4.

Supplementary Material 5.

Supplementary Material 6.

Supplementary Material 7.

## Data Availability

All the data supporting the findings of this work are available within the article and its Supplementary information files.

## Abbreviations

MNB: Mediastinal neuroblastoma
ICIs: Immune checkpoint inhibitors
NB: Neuroblastoma
TILs: Tumor Infiltrating lymphocytes
PD1: Programmed cell-death protein 1
BTLA: B- and T-lymphocyte attenuator
CTLA4: Cytotoxic T lymphocyte-associated molecule 4
TIGIT: T cell Ig and ITIM domain
LAG: Lymphocyte-activation gene 3
TIM3: T-cell immunoglobulin and mucin-domain containing 3
GSLs: Glycosphingolipids
LacCer: Lactosylceramide
OS: Overall survival
PFS: Progression-free survival
GEO: Gene expression omnibus
NMF: Non-negative matrix factorization
GO: Gene ontology
KEGG: Kyoto encyclopedia of genes and genomes
PCA: Principal component analysis
GSEA: Gene set enrichment analysis
DEGs: Differential expressed genes
ssGSEA: Single Sample Gene Set Enrichment Analysis
RIN: RNA integrity number
PBMCs: Peripheral blood mononuclear cells
ATCC: American Type Culture Collection
FBS: Fetal bovine serum
IHC: Immunohistochemistry
IF: Immunofluorescence
EV: Empty vector
CM: Conditional Medium
PI: Propidium iodide
ChIP: Chromatin immunoprecipitation
ADCC: Complement-mediated elimination and antibody-dependent cell-mediated cytotoxicity
Siglec-10: Sialic acid-binding immunoglobulin-like lectin
LRH-1: Liver receptor homolog 1
LXR: Liver X receptor (LXR)
PPAR: Peroxisome proliferator-activated receptor
ITIMs: Intracellular immunoreceptor tyrosine-based inhibitory motifs
TBP: TATA-binding protein
PMA: Phorbol 12-myristate 13-acetate
