# Supplementary material for: EDF1 accelerates ganglioside GD3 accumulation to boost CD52-mediated CD8+ T cell dysfunction in neuroblastoma
Source: J Exp Clin Cancer Res. 2025 Feb 4;44:36. doi: 10.1186/s13046-025-03307-9 (PMC11792593; doi:10.1186/s13046-025-03307-9)
Supplement: Supplementary file 6 — Supplementary Material 6. [file 13046_2025_3307_MOESM6_ESM.docx]

**Supplementary figures and figures legends**

**
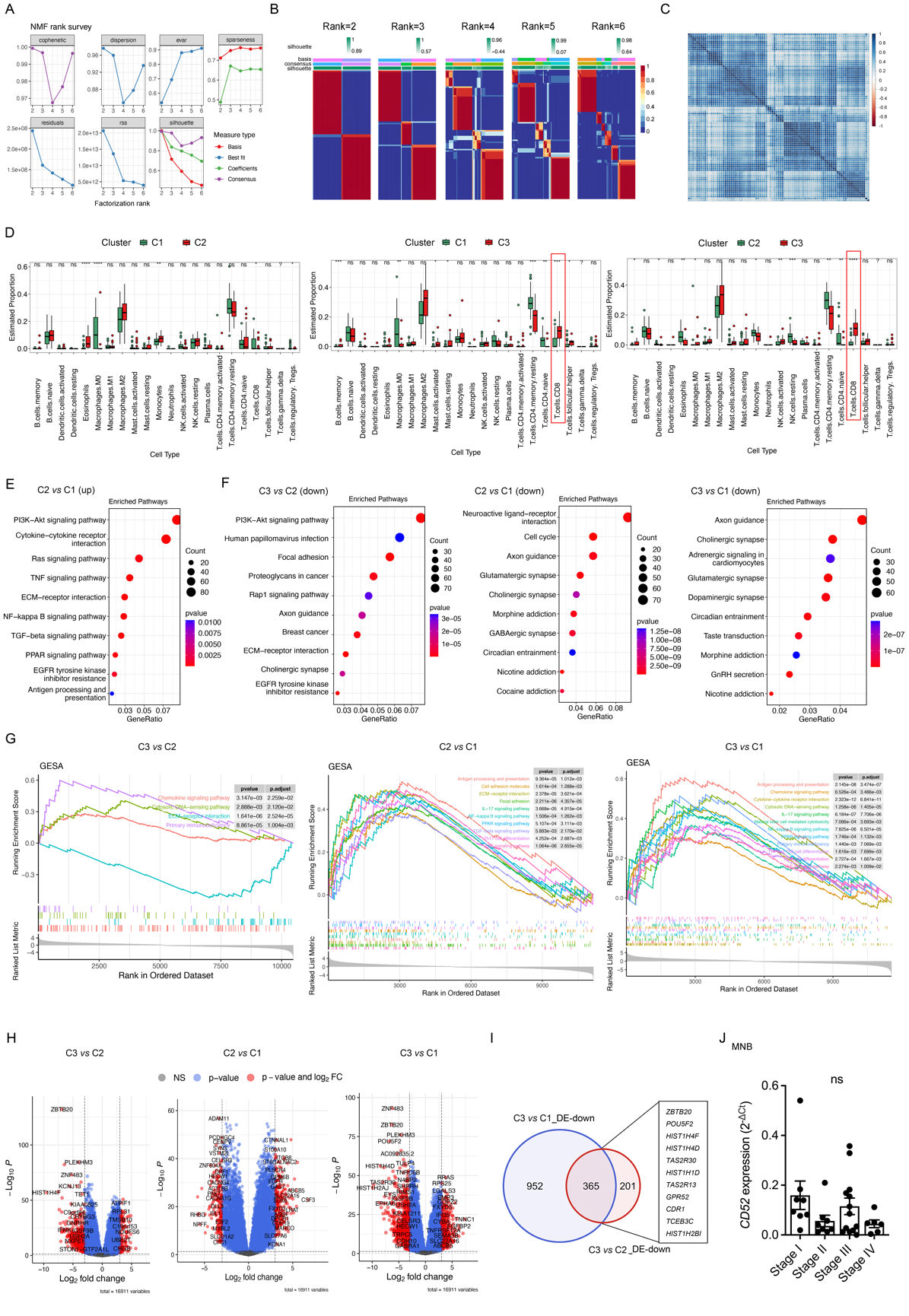
**

**Fig. S1 Bioinformatic analysis highlights the immune-inflamed cluster in MNB.** **(A)** The rank survey from NMF analysis of 89 surgically resected MNB tumors. **(B)** Heatmap of the consensus matrix in the MNB samples (Rank = 2 to 6). **(C)** Correlation analysis divides the MNB samples into three clusters. **(D)** Boxplot of the enrichment scores of major immune cell types across the three clusters by Cibersort classification. **(E)** KEGG enrichment analysis of upregulated DEGs in C2 *v.s.* C1 (log2fold change > 2 & *P* < 0.05). **(F)** KEGG enrichment analysis of downregulated DEGs from three pairs of comparisons (log2fold change < -2 & *P* < 0.05). **(G)** GSEA enrichment analysis of total DEGs from three pairs of comparisons (*P* < 0.05). **(H)** Volcano plots of significant DEGs by a cutoff value of |log2fold change| > 4. **(I)** Venn diagram of downregulated DEGs in C3 *v.s.* C2 and C3 *v.s.* C1. **(J)** mRNA expression of *CD52* in each stage of MNB. The data in J represent the mean ± SD.

**
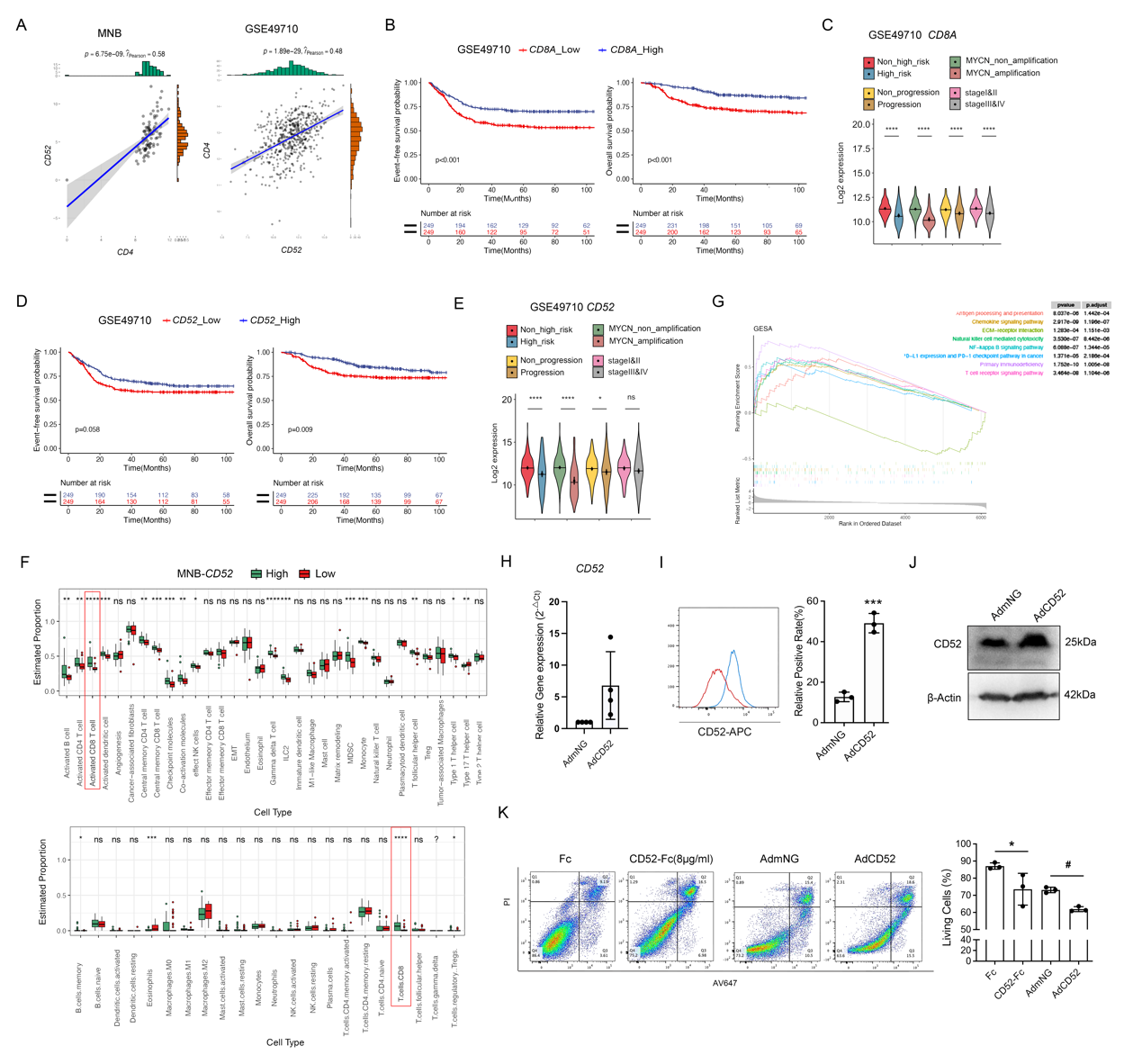
**

**Fig. S2 The prognostic feature of CD52 in NB tumors largely depends on CD8^+^ T cell infiltration. (A)** Correlation analysis showing the expression of *CD52* and *CD4* in the MNB tumors and NB dataset (GSE49710). **(B, D)** EFS and OS of *CD8A* **(B)** and *CD52* **(D)** in NB tumors (GSE49710). **(C, E)** Log2 expression of *CD8A* **(C)** and *CD52* **(E)** across distinct clinicopathological characteristics of NB tumors (GSE49710). **(F)** Boxplot of the enrichment scores in major immune components by ssGSEA (top panel) and Cibersort classification (bottom panel) when stratifying into CD52^high^ and CD52^low^ MNB tumors. **(G)** GSEA enrichment analysis of total DEGs between CD52^high^ and CD52^low^ subsets. **(H, I, J) q**RT-PCR, flow cytometry and immunoblotting analysis were applied to verify CD52 expression on CD8^+^ T cells followed by adenovirus transfection with full-length CD52. β-Actin was used as a loading control. **(K)** Apoptosis assay of activated CD8^+^T cells exposed to CD52-Fc treatment or the full-length overexpression (AdCD52) procedure. ^*^*P* & ^#^*P* < 0.05, ^***^*P* < 0.001, ^****^*P* < 0.0001. The data in H, I, K represent the mean ± SD (n = 3). Fc and AdmNG (ADV-mNeonGreen) are controls for CD52-Fc and AdCD52, respectively.

**
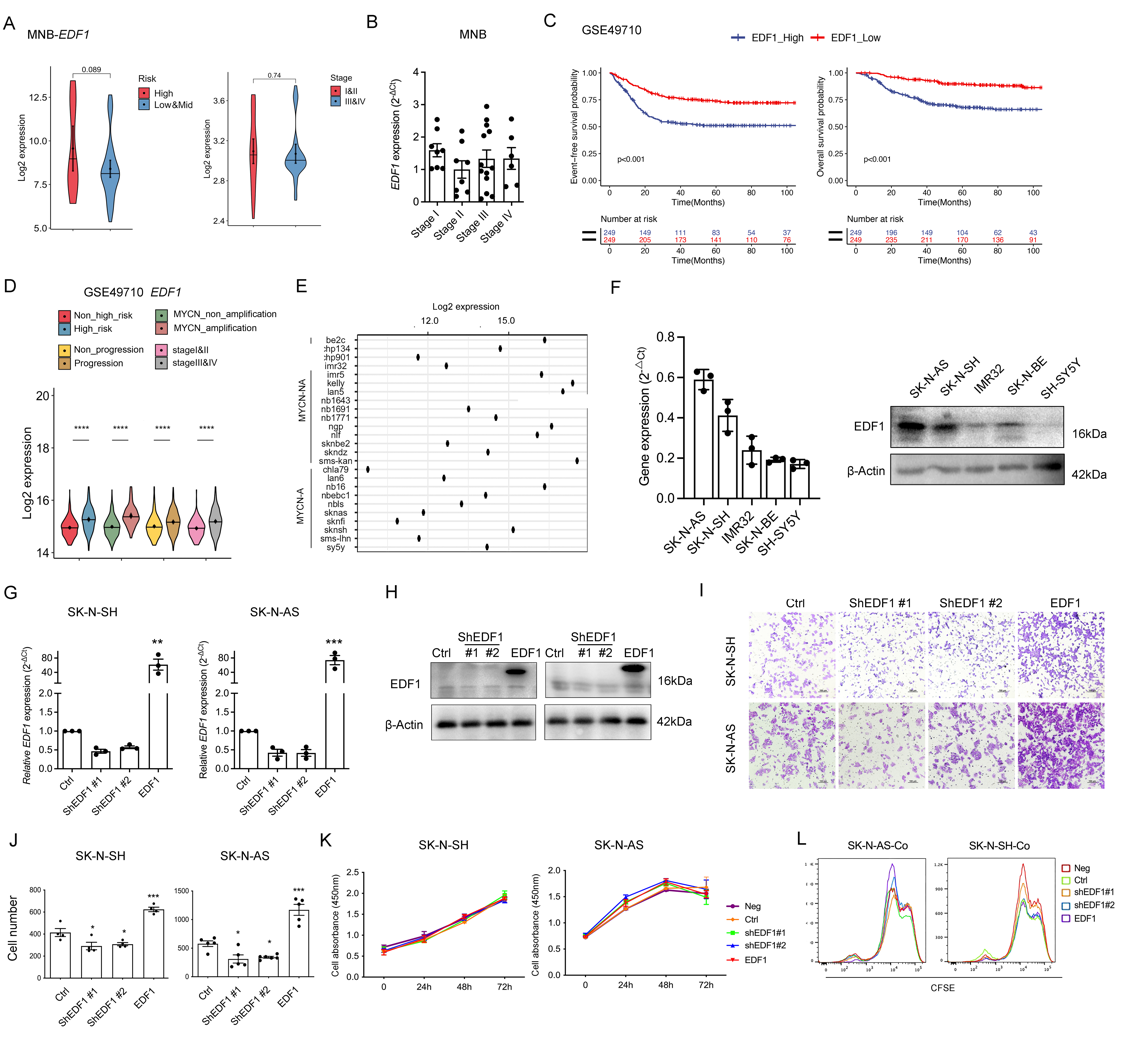
**

**Fig. S3 High EDF1 level exerts pro-migration activity and predict poor survival in NB tumors. (A)** Log2 expression of *EDF1* in newly diagnostic MNB transcriptional matrix(n=68), according to risk and stage stratification. **(B)** RT-PCR analysis of *EDF1* in each stage of MNB tumors. **(C)** The EFS and OS of *EDF1* in NB tumors (GSE49710). **(D)** Log2 expression of *EDF1* across distinct clinicopathological characteristics of NB tumors (GSE49710). ^****^*P* < 0.0001. **(E)** Log2 expression of *EDF1* in both MYCN-A- and MYCN-A-derived NB cell lines (GSE19274). **(F) q**RT-PCR (left panel) and immunoblotting (right panel) analyses of *EDF1* in NB cell lines. **(G-H)** The mRNA and protein levels of *EDF1* in NB cell lines after transfection were detected by qRT‒PCR and immunoblot analysis. β-Actin was used as a loading control for immunoblotting. **(I)** Transwell assay of NB cells transfected with sh#EDF1 or full-length plasmid. Statistical analysis is shown in **(J)**. **(K)** The proliferation rate of NB cells transfected as indicated was assessed by CCK-8 assay. **(L)** The proliferation rate of CD8^+^ T cells cocultured with EDF1-altered NB cells was visualized by CFSE staining. Ctrl in G-L stands for the average of undistinguishable controls of scrambled sequence (for Sh-EDF1) and empty vector (for EDF1 overexpression). The data in F, G, J-K represent the mean ± SD (n = 3). ^*^*P* < 0.05, ^**^*P* < 0.01, ^***^*P* < 0.001.


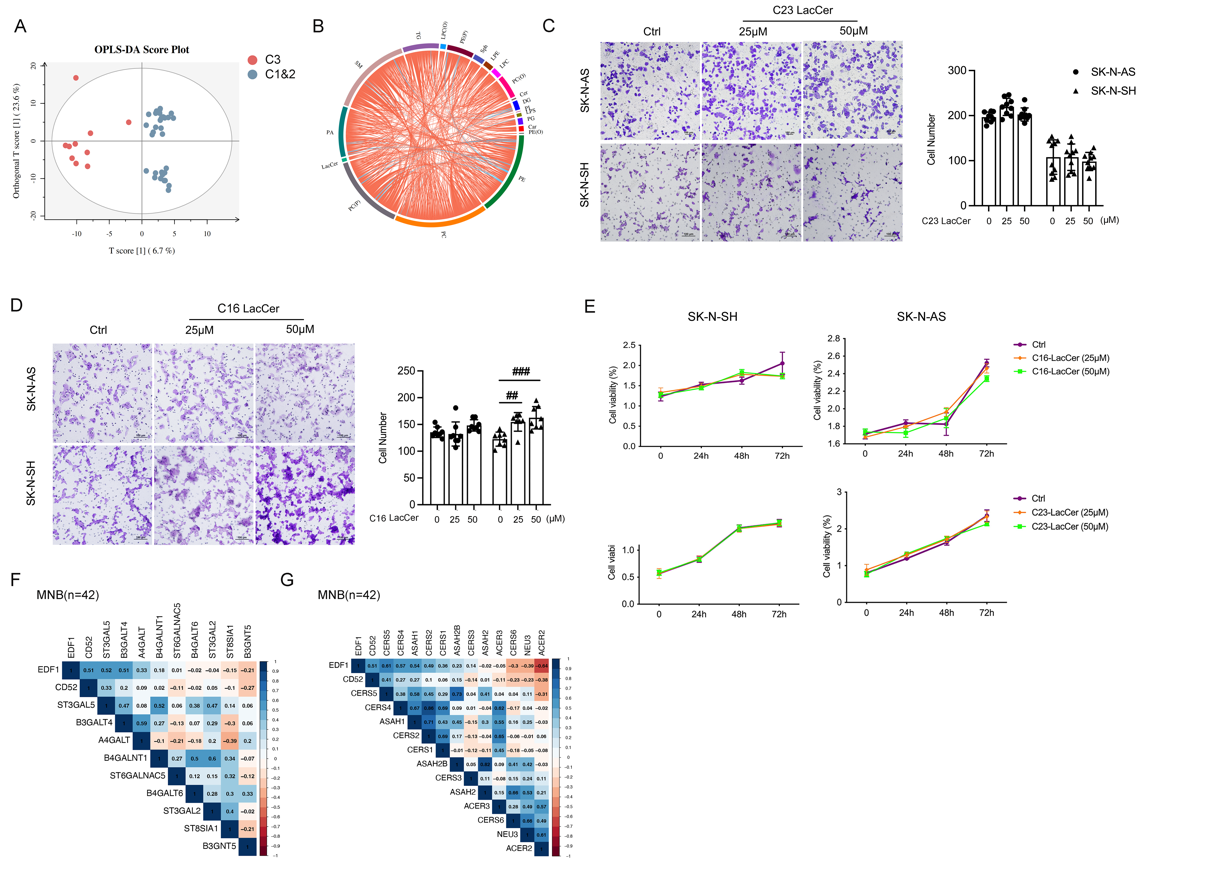


**Fig. S4 Identification of lipidomic metabolites in MNB by lipidomic analysis. (A)** Lipidomic metabolites from the three clusters in MNB tumors (n = 42) were discriminated by PCA. **(B)** The circos plot of interconnections among lipidomic metabolites in MNB tumors. **(C, D)** Transwell assay of NB cells induced by exogeneous C23 **(C)** and C16 LacCers. Statistical analysis is shown in **(D)**. **(E)** The proliferation rate of NB cells exposed to exogeneous C23 and C16 LacCers was measured by CCK8 assay. **(F)** Correlation plots among EDF1, CD52 and synthases for gangliosides. **(G)** Correlation plots among EDF1, CD52 and synthases for LacCers in MNB tumors (n=42). The data in C-E represent the mean ± SD (n = 3). ^*^*P* < 0.05, ^##^*P* < 0.01, ^###^*P* < 0.001.


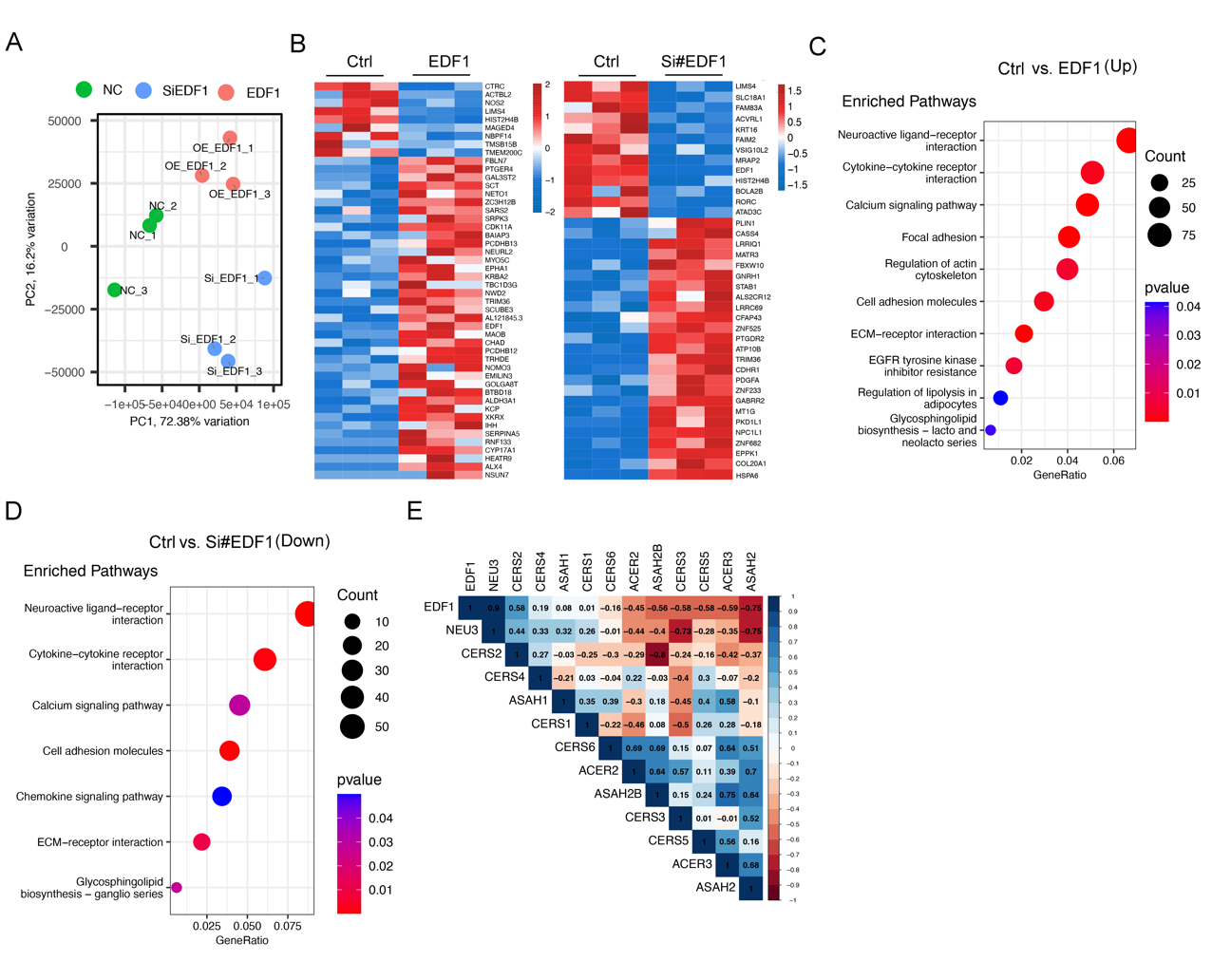


**Fig. S5 EDF1 engages in LacCer-ganglioside metabolism. (A)** PCA of the RNA sequencing matrix involving Ctrl, EDF1-overexpressing and EDF1-knockdown SK-N-AS cells. **(B)** Heatmaps of DEGs in EDF1 *vs.* Ctrl, and si#EDF1 *vs.* Ctrl, separately (fold change > 3 & *P* < 0.05). **(C, D)** KEGG analysis of genes upregulated in EDF1 *vs* Ctrl or downregulated in siEDF1#1 *vs* Ctrl. **(E)** Correlation plots among EDF1 and synthases for LacCers in an RNA sequencing matrix involving Ctrl, EDF1-overexpressing and EDF1-knockdown SK-N-AS cells. All the data above are presented as the mean ± SD (n = 3).


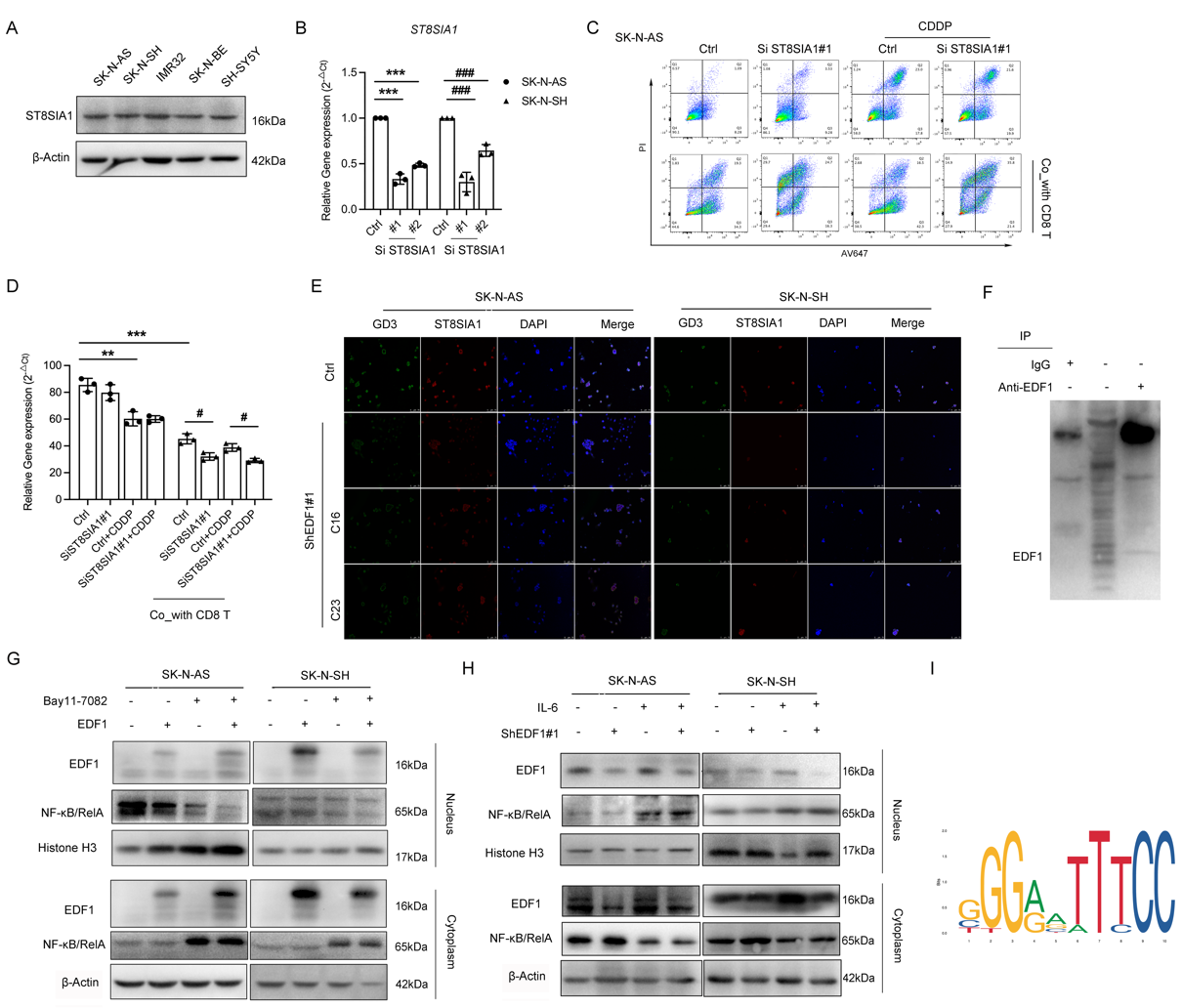


**Fig. S6 EDF1 is responsible for increased GD3 content induced by LacCer through binding to NF-κB/RelA and rarely affects the cellular distribution of NF-κB/RelA. (A)** Immunoblotting analyses of ST8SIA1 in NB cell lines. β-Actin was used as a loading control. **(B)** RT-PCR analysis of *ST8SIA1* in NB cells transfected with siRNAs. The data represent the mean ± SD (n = 3). **(C, D)** Apoptosis and statistical assay of SK-N-AS treated as indicated. The data in B and D represent the mean ± SD (n = 3). ^*^*p* & ^#^*p* < 0.05, ^**^*p* & ^##^*p* < 0.01, ^***^*p* & ^###^*p* < 0.001. **(E)** Representative immunofluorescence staining images of ST8SIA1 (green) and GD3 (red) treated as indicated. DAPI (blue) was used to stain nuclei. **(F)** Coimmunoprecipitation (Co-IP) assays were performed to verify the specificity of the anti-EDF1 antibody. **(G, H)** Immunoblot analysis of NF-κB/RelA and EDF1 expression in the cytoplasm and nucleus of NB cells treated as indicated. β-Actin and histone H3 were used as loading controls separately in the cell cytoplasm and nucleus. **(I)** The predicted binding site of NF-κB/RelA in the ST8SIA1 promoter, according to JASPAR database prediction.

**
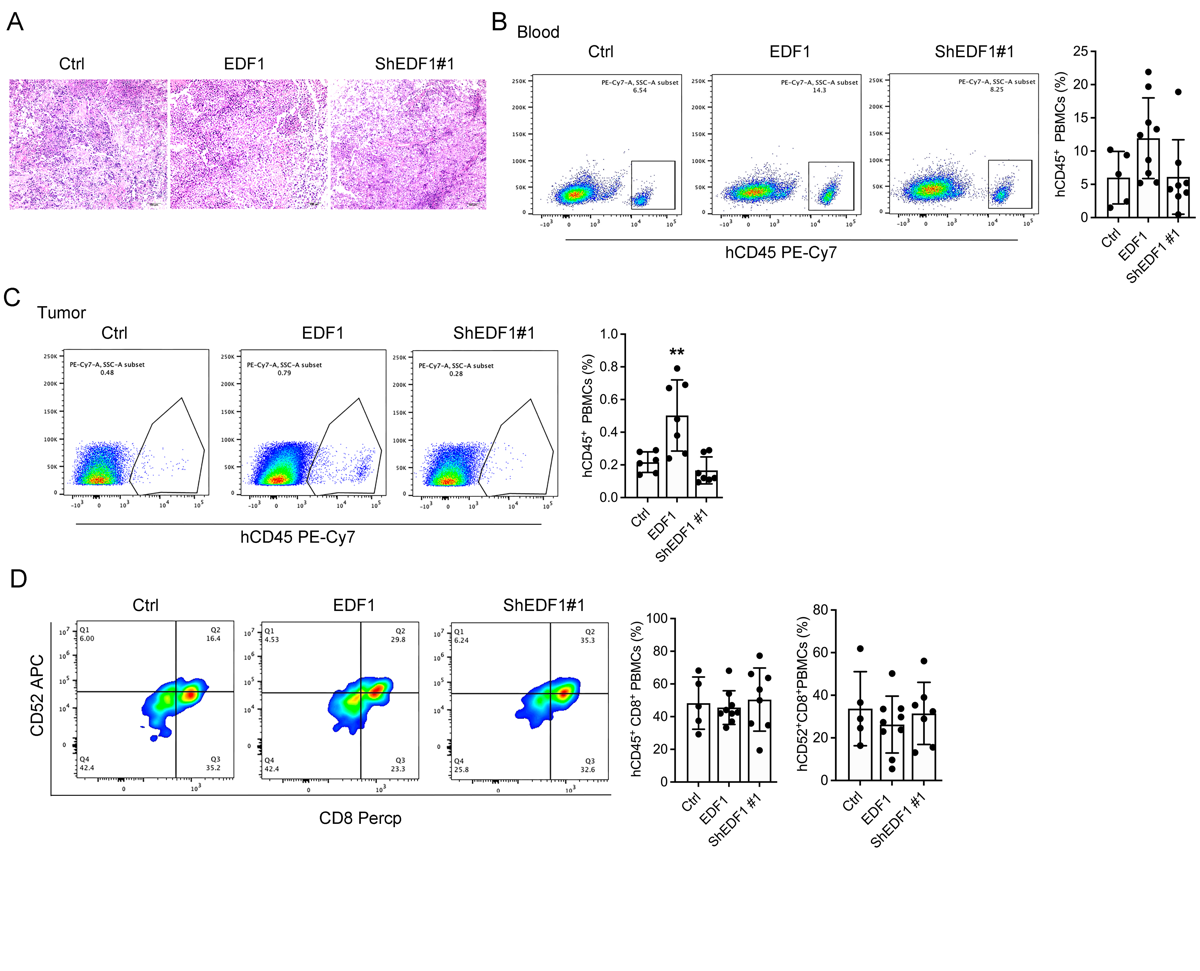
**

**Fig. S7 EDF1-altered NB tumors educate human CD8^+^ T cells *in vivo*. (A)** Histological analyses in each group of humanized orthotopic NB tumors. **(B, C)** Representative flow cytometric plots of human CD45^+^ lymphocytes in blood **(B)** and tumors **(C)** from each group. **(D)** Representative flow cytometric plots of human CD45^+^ lymphocytes isolated from tumors in each group and further staining with human CD8 and CD52. The above data are presented as the mean ± SD (n = 5).
